# 16S amplicon sequencing of microbial communities in enriched and non-enriched sediments of non-volcanic hot spring with temperature gradients

**DOI:** 10.7717/peerj.10995

**Published:** 2021-04-02

**Authors:** Muhammad Yasir, Arooj K. Qureshi, Esam I. Azhar

**Affiliations:** 1 Special Infectious Agents Unit, King Fahd Medical Research Center, King Abdulaziz University, Jeddah, Saudi Arabia; 2 Medical Laboratory Technology Department, Faculty of Applied Medical Sciences, King Abdulaziz University, Jeddah, Saudi Arabia

**Keywords:** Hot spring, 16S amplicon sequencing, Thermophile, Tatta Pani, Culture-dependent, Enrichment

## Abstract

Microorganisms in geothermal springs can offer insights into the fundamental and applied study of extremophiles. However, low microbial abundance and culturing requirements limit the ability to analyze microbial diversity in these ecosystems. In this study, culture-dependent and culture-independent techniques were used to analyze sediment samples from the non-volcanic Tatta Pani hot springs in district Poonch of Azad Kashmir. Microbial composition, temperature gradient, and enrichment effects on rare taxa were evaluated. In total, 31 distinct bacterial phyla and 725 genera were identified from the non-enriched Tatta Pani hot spring sediment samples, and 33 distinct bacterial phyla and 890 genera from the enriched sediment samples. Unique phyla specimens from the enriched samples included Candidatus Cloacimonetes, Caldiserica, and Korarchaeota archaea. The enriched samples yielded specific microbiota including 805 bacteria and 42 archaea operational taxonomic units with 97% similarity, though decreased thermophilic microbiota were observed in the enriched samples. Microbial diversity increased as temperature decreased. Candidate novel species were isolated from the culture-dependent screening, along with several genera that were not found in the 16S amplicon sequencing data. Overall, the enriched sediments showed high microbial diversity but with adverse changes in the composition of relatively dominant bacteria. Metagenomic analyses are needed to study the diversity, phylogeny, and functional investigation of hot spring microbiota.

## Introduction

Hot springs are geothermal ecosystems with diverse thermophilic bacteria (Inskeep et al., 2010; Jiang & Takacs-Vesbach, 2017). These bacteria have evolved in several ways, such as selective pressurizing of biochemical machinery (e.g., lipids, enzymes, and proteins), to adapt to harsh physiochemical parameters (Swingley et al., 2012; Wang, Cen & Zhao, 2015). Recent findings from studies of thermophilic microorganisms indicate their potential value in drug development, industrial applications, bioremediation, and cellular studies (Schröder et al., 2014; Shah et al., 2015; Verma et al., 2015). Moreover, the characterization of microorganisms from such environments offers great insights into the origins and evolution of primitive life (Sakai & Kurosawa, 2016).

Metagenomic studies revealed diverse microbial communities in hot springs dominantly comprised of bacterial phyla Chloroflexi, Proteobacteria, Aquificae, Firmicutes, Actinobacteria, Deinococcus-Thermus, and Bacteroidetes (Inskeep et al., 2013; Jiang & Takacs-Vesbach, 2017; Wilkins et al., 2019). From archaea, Crenarchaeota, Euryarchaeota, and Thaumarchaeota are commonly found in hot springs’ microbiota (Barns et al., 1994; Poddar & Das, 2018; Wilkins et al., 2019). Volcanic geothermal areas in the United States, Iceland, New Zealand, Russia, and China have been widely studied, revealing substantial variation in the physicochemical properties that shape microbial communities (Boyd et al., 2010; Colman et al., 2019; Cousins et al., 2018; Payne et al., 2019; Power et al., 2018; Ward et al., 2017; Wilkins et al., 2019; Zhang et al., 2018). For instance, γ-Proteobacteria, Thermotogae, and Euryarchaeota are the dominant residents of the Mutnovsky hot spring in the southern Kamchatka Peninsula in Russia. This hot spring has a temperature 70 °C and pH between 3.5 and 4.0. On the same peninsula, the Uzon Caldera hot spring houses β-Proteobacteria, γ-Proteobacteria, and Thermodesulfobacteria at a temperature of 81 °C and pH between 7.2 and 7.4 (Daniel, 2013).

The microbial communities of non-volcanic hot springs have not been largely evaluated, possibly because of their scarcity. According to the literature, these hot springs usually house bacterial phyla including Firmicutes, Proteobacteria, Cyanobacteria, Bacteroidetes, Actinobacteria, Chloroflexi, Acidobacter, Deinococcus-Thermus, and Nitrospira, along with the archaeal phyla Crenorchaeota and Euryarcheaota (Amin et al., 2017). Chan et al. (2017) observed a similar distribution of dominant phyla in six non-volcanic hot springs of different physicochemical properties from Malaysia, commonly comprised of Aquificae, Chlorobi, Thermotogae, Proteobacteria, and Firmicutes. But variation was observed in overall microbial communities composition in sites (Chan et al., 2017). Previous studies identified temperature as a main factor in shaping microbial community composition in hot springs compared to biogeography and other environmental parameters such as pH, and water chemistry (Jiang & Takacs-Vesbach, 2017; Miller et al., 2009; Payne et al., 2019; Zhang et al., 2018). But most of the studies did not differentiate the abundant and rare bacterial taxa in the total bacterial community. The abundant taxa contribute major in biomass, but minor in microbial diversity, whereas the rare taxa contribute minor in biomass but major in microbial diversity (Yamamoto et al., 2019; Zhang et al., 2018). The rare taxa respond differently to environmental factors and play important role in biogeochemical cycles (Jousset et al., 2017; Yamamoto et al., 2019).

Geothermal springs are mainly of non-volcanic origin in Pakistan and the Indian subcontinent (Amin et al., 2017; Poddar & Das, 2018). Pakistan’s position over the intersections of tectonic plates of the sub-continent make it one of the richest geothermally active areas in the world. Among its active fault sites, the Main Boundary Thrust, Main Mantle Thrust, and Main Karakorum Thrust (Bakht, 2000; Zaigham, 2005) generate three main geothermal sites: the Chagai volcanic arc (volcanic hot springs in Baluchistan), the Indus basin margin (non-volcanic hot springs in Sindh), and the Himalayan collision course (non-volcanic sites including Sassi, Mushkin, Garam Chashma, and Tatta Pani) (Bakht, 2000; Sheikh, 2009; Zaigham, Nayyar & Hisamuddin, 2009). Tatta Pani hot spring is situated on the right bank of the Poonch River in District Poonch. The recharge source is meteoric water, and residence time is calculated about 40 years based on isotopic and chemical analysis (Anees, Shah & Qureshi, 2015). Mountain rainwater seeps through fault lines and then is likely heated by cooling magma or hot rocks. This heated water rises to the surface and mixes with shallow, non-thermal groundwater to form a hot stream (Anees, Shah & Qureshi, 2015; Baioumy et al., 2015). Studies reported thermophilic bacteria from Tatta Pani hot spring used mainly culture-dependent approaches (Amin et al., 2016; Jadoon et al., 2014; Javed et al., 2012).

In this study, we used 16S amplicon sequencing to explore the taxonomic diversity of abundant and rare microbial communities in Tatta Pani hot spring. A culture enrichment approach was adopted to enhance the hot spring captured microbial diversity using specific media in the laboratory environment. Moreover, the enrichment effect on overall microbial community composition and temperature influence was identified from a comparison between non-enriched Tatta Pani (TP) and enriched Tatta Pani (ETP) sediment samples collected at different temperatures.

## Materials and Methods

### Sampling

**Figure 1:**
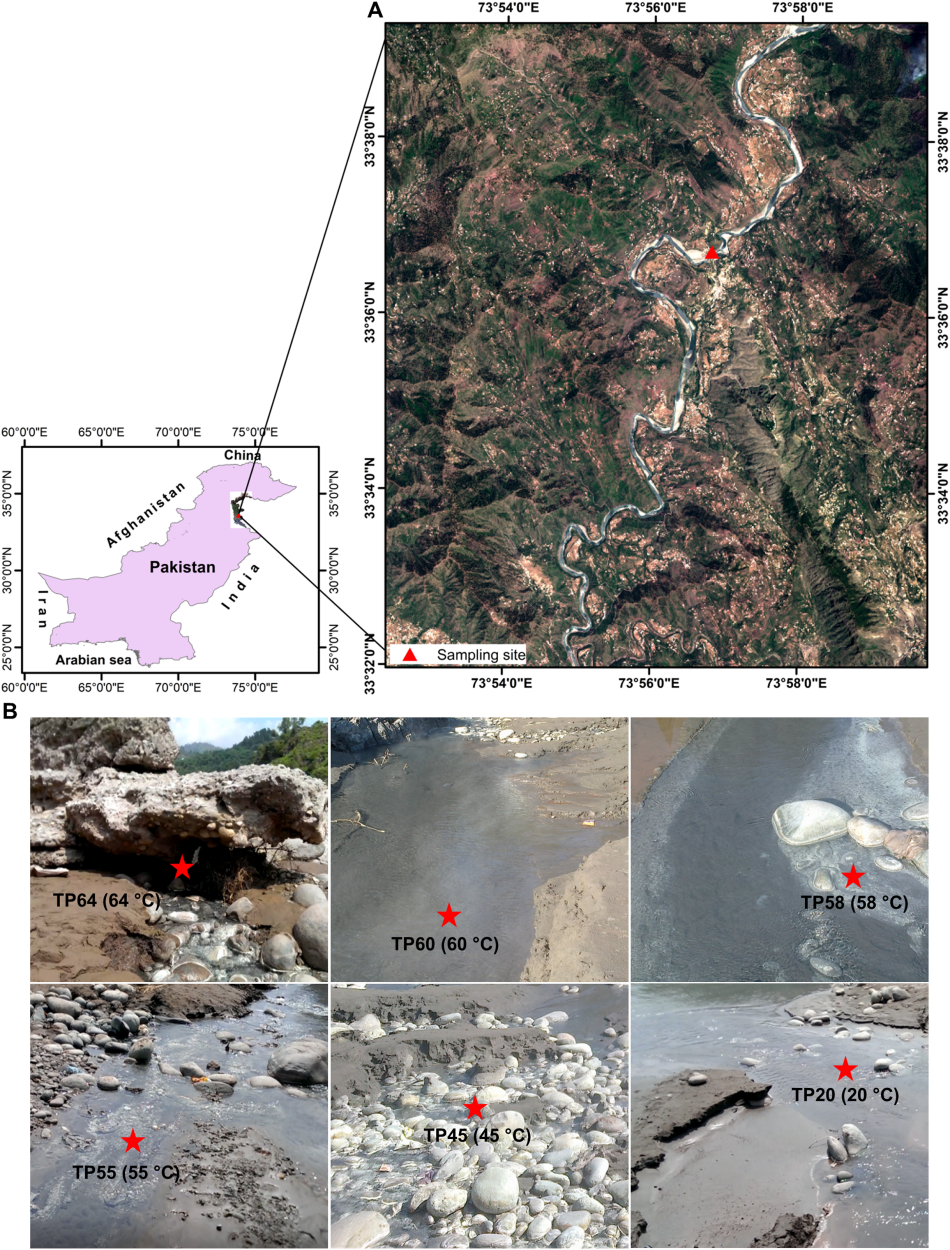
Map and images of the Tatta Pani hot spring. (A) ****The sample location is overlaid on the Sentinel-2 image obtained through free public access from Sentinel-2 multispectral satellite images provided by the European Space Agency (© European Space Agency). (B) Images of the sample collection sites at different temperatures.

We collected six sediment samples at Tatta Pani hot springs, including one from the hot spring source and five from downstream locations along a temperature gradient (Figs. 1A and 1B; Table S1). The samples were collected using long, sterilized spatulas and then placed in 50-mL Falcon tubes and stored at 4 °C during transportation. The samples were stored at −20 °C for 1 week before further processing for DNA extraction. We processed total 12 samples: six non-enriched Tatta Pani (TP) and six enriched Tatta Pani (ETP) subsamples (Table S1). No specific permission was required for sampling the studied hot spring that was not privately owned or protected in any way. The hot spring was not part of a national park or reserve.

### Enrichment of the samples

Microbial enrichment of sediment samples was performed using 10 g from each sample in 250 mL of customized media broth (Table S2) containing 50% of 0.2 μm membrane filter (Fisher Scientific, Waltham, MA, USA) sterilized hot spring water in an autoclaved 1,000-mL flask (Pyrex, Germany). Each flask then was incubated for 1 week at 100 rpm, maintaining the respective temperature of the sample as at the site of collection. The enriched cultures were centrifuged at 7,000 g for 15 min. The pellets were used for genomic DNA extraction.

### 16S amplicon sequencing and data processing

Genomic DNA was isolated from 300 mg of both original and enriched sediment samples using the PowerSoil® DNA Isolation Kit (MO BIO Laboratories, Carlsbad, CA, USA) following the manufacturer protocol. Amplicon sequencing was performed targeting the V4 variable region using universal primers 515F and 806R according to previously described methodology (Yasir et al., 2015). Briefly, DNA concentration was checked using the Qubit system (Invitrogen, Waltham, MA, USA). Sequencing adapters and dual-index barcodes were joined to the sequence-reads using a limited PCR cycle. After purification via Agencourt AMPure beads (Agencourt, Beverly, MA, USA), the libraries were normalized according to the Nextera XT protocol. Following the manufacturer’s protocol, the samples were loaded into a single flow cell for sequencing on the MiSeq system (Illumina, Inc., San Diego, CA, USA).

Paired-end FASTQ files were assembled using PANDAseq (Masella et al., 2012). Filtration of the sequence reads was performed, including cleaning of primers and barcode regions. All reads with ‘N’ and size <200 bp were removed. Chimaeras and singleton reads were deleted. Sequence reads of high quality were grouped into operational taxonomic units (OTUs) with ≥97% sequence similarity using QIIME 1.9 (Caporaso et al., 2010). Finally, a curated database derived from GreenGenes, the Ribosomal Database Project RDPII, and the National Center for Biotechnology Information was created using BLASTn for taxonomic assignment of the OTUs. The sequence data of this study was submitted to the European Nucleotide Archive under accession nos. SAMEA6812218–SAMEA6812229.

### Culture-dependent screening

The hot spring source sample from 64 °C (TP64) was processed for culture-dependent screening using a previously described, modified approach to increase recovery of slow-growing bacteria (Yasir et al., 2019). Briefly, 10 g of sediment sample was dispersed in 90 mL of normal saline and shaken for 45 min at 200 rpm. Sample was serially diluted in 10 mL normal saline (10^1^ to 10^4^), and a 100 μL aliquot of each dilution was spread in low-nutrient media (Table S2) supplemented with 1.5% agar. The culture plates were incubated at three temperatures (37 °C, 45 °C, and 60 °C). Colonies were purified by sub-culturing and preserving in 15% glycerol suspension at −80 °C. Biosafety level-2 cabinets were used to avoid contamination and risk factors.

A high-throughput MALDI-TOF mass spectrometry-based MALDI Biotyper system (Bruker Daltonics, Billerica, MA, USA) was used to identify purified isolates, following the procedures described in our previous study (Angelakis et al., 2014). A species was considered to be correctly identified if the spectrum presented a score of ≥1.9 (Angelakis et al., 2014). Isolates with scores <1.9 were identified by 16S rRNA gene sequencing using universal primers 27F and 1492R (Yasir et al., 2009). The isolates were identified from the blast analysis of 16S rRNA gene sequences against the EzBioCloud nucleotide database (Kim et al., 2012). A threshold similarity of <97% was used to define new bacterial candidate species (Tindall et al., 2010). Phylogenetic tree of 16S rRNA gene sequence of candidate novel isolate with closely related type strains was constructed using Maximum likelihood method based on the Tamura-Nei model with 1,000 bootstrap value using MEGA X software (Kumar et al., 2018).

### Statistical analysis

Data normality was ascertained using a Kolmogorov–Smirnov D test. Kruskal–Wallis H and Mann–Whitney tests (for non-normal data) were performed to observe significantly different bacterial taxa among the samples. The Calypso 8.84 and Past 4.01 tools were used for alpha and beta diversity analyses. Cytoscape (3.7.2) was used for networking analyses. SPSS version 22 (IBM, Armonk, NY, USA) was used for statistical analysis.

## Results

### Diversity analysis

The hot spring source temperature was 64 °C, and all collected samples were weakly alkaline in pH (Table S1). We obtained 0.88 million trimmed and high-quality sequence reads from the TP and ETP samples. Sequence reads were classified into 2,083 OTUs with 97% similarity and assigned to microbial domains. In both TP and ETP samples, 37 total phyla were detected, including 33 bacteria and 4 archaeal phyla (Fig. S1; Table S3). Among those, 20 bacterial phyla and 3 archaeal phyla were common to both groups. Moreover, 292 families, 1,006 genera, and 1,997 OTUs of bacteria at the species level were retrieved from the all Tatta Pani hot spring samples. Among archaea, 27 families, 53 genera, and 86 OTUs at the species level were detected.

**Figure 2:**
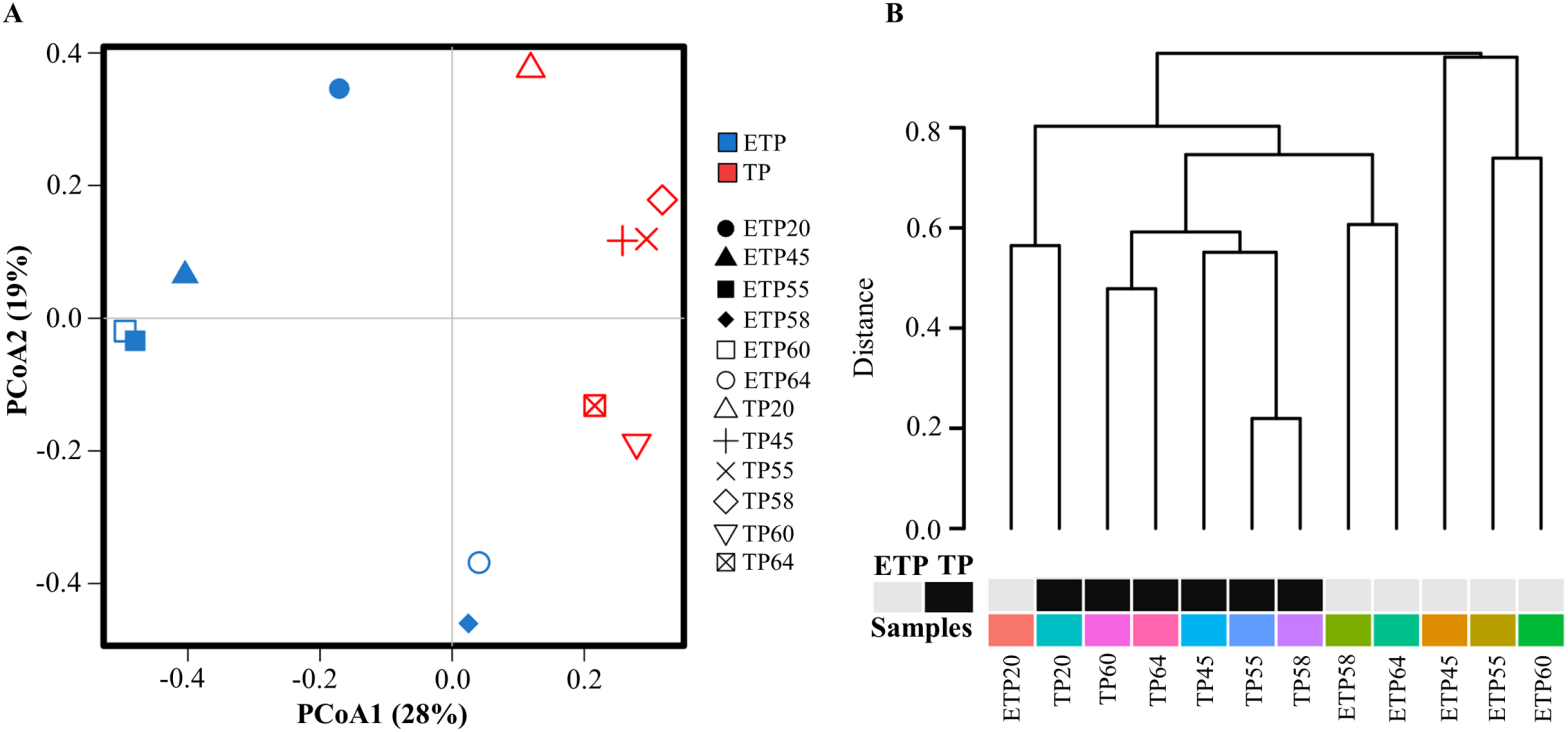
Multivariate analysis of the samples from Tatta Pani hot spring. (A) Principal coordinate analysis and (B) hierarchical clustering using Bray-Curtis dissimilarity analysis revealed a shift in the microbial community with temperature and enrichment changes. The alphabetical letters in the sample name represent the sample group, and the numeric value indicates the temperature in centigrade. TP, sediment samples from Tatta Pani hot spring; ETP, enriched sediment samples from Tatta Pani hot spring.

Multivariate principal coordinate analysis and cluster analysis of OTUs revealed a shift in microbial abundance and diversity between the TP and ETP samples (Figs. 2A and 2B). The TP samples showed more clustering than the ETP samples, except for TP20, which was close to ETP20 (Fig. 2A). We used Bray-Curtis dissimilarity analysis to assess hierarchical clustering in the TP and ETP samples. We found separate clusters in both groups, and the TP samples had closely linked clusters than the ETP group suggest that microbial community changed with the enrichment compared to the no-enriched sample of the same temperature (Fig. 2B). A Pearson’s correlation analysis of species-level OTUs revealed that diversity decreased significantly as temperature increased in both TP (*p* = 0.003; *r* = −0.8) and ETP samples (*p* = 0.011; *r* = −0.9) (Figs. S2A and S2B).

### Phyla analysis

**Figure 3:**
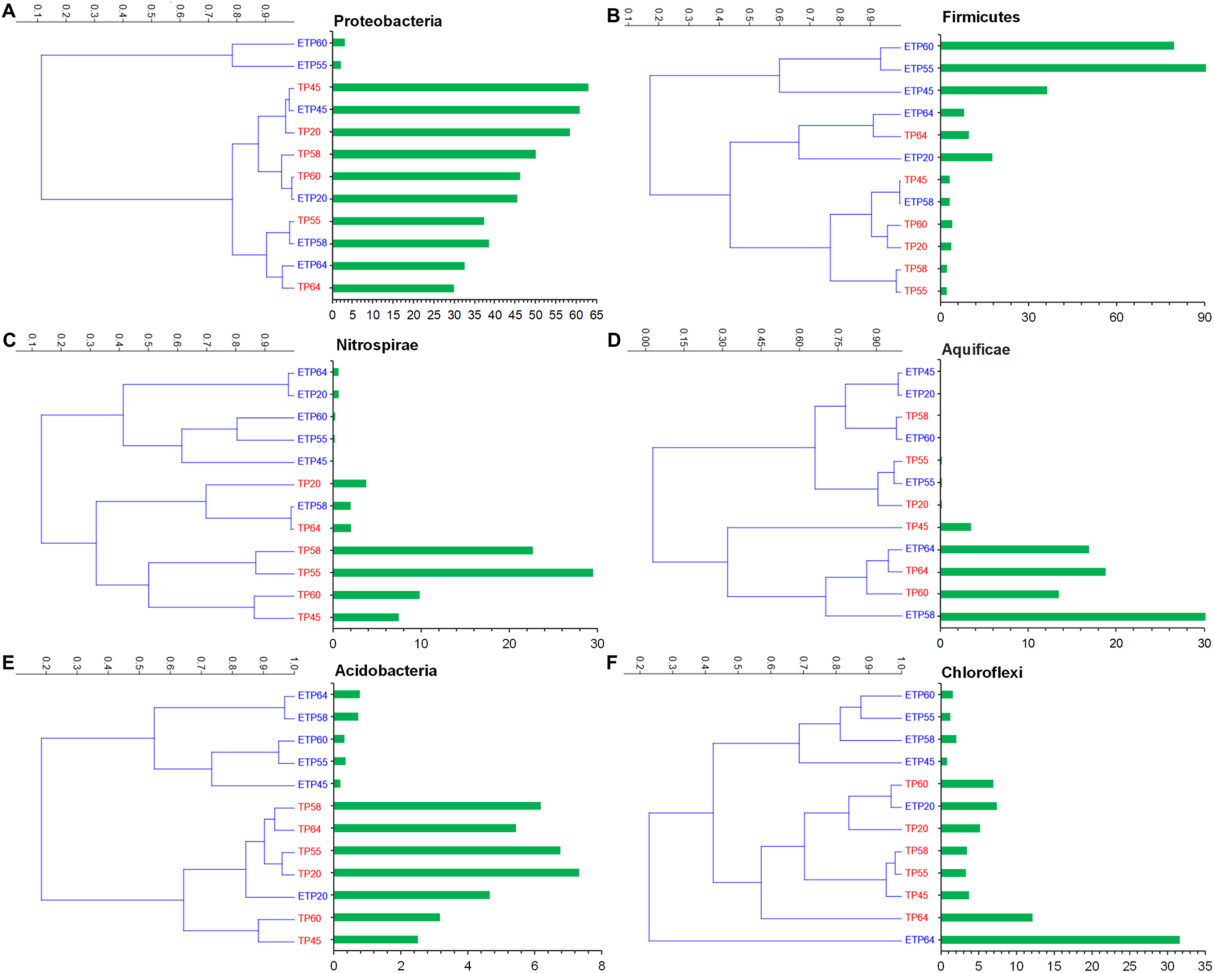
Hierarchical clustering of six relatively abundant phyla in the enriched and nonenriched samples from Tatta Pani hot spring, using the unweighted pair group method with arithmetic mean tree in the Bray–Curtis analysis. The bar chart represents the relative abundance of phyla in percentages (A) Proteobacteria, (B) Firmicutes, (C) Nitrospirae, (D) Aquificae, (E) Acidobacteria, and (F) Chloroflexi. The alphabetical letters in the sample name represent the sample group, and the numeric value indicates the temperature in centigrade. TP, sediment samples from Tatta Pani hot spring; ETP, enriched sediment samples from Tatta Pani hot spring.

The most commonly observed phyla in both the TP and ETP groups included Proteobacteria, Firmicutes, Nitrospirae, Aquificae, and Chloroflexi (Figs. 3A–3F). Proteobacteria were more abundant in the TP samples (30–62.9%), particularly the TP45 (62.9%) and TP20 (58.4%) samples (Fig. 3A) than in the ETP samples (30.5 ± 23.6%). Variation in relative abundance was observed between the enriched and non-enriched samples from the same temperature (Fig. 3A). Substantially higher Firmicutes relative abundance was observed in the ETP samples, particularly ETP55 (90.8%) and ETP60 (79.5%), than in the TP samples (2.1–9.6% Fig. 3B). Nitrospirae were found at relatively high abundance (2.0–29.5%) in the TP samples, particularly TP55 (Fig. 3C), compared to 0.1–2.0% in the ETP samples.

Phylum Aquificae showed high abundance (≥13.5%) in the high-temperature (≥60 °C) TP samples and in the 64 °C and 58 °C ETP samples, compared with ≤3.5% in the other temperature samples from both groups (Fig. 3D). Acidobacteria were found at relatively higher abundance (2.5–7.3%) in the TP samples than in the ETP samples (<1%), except for ETP20 (4.7%), that made a cluster with TP samples (Fig. 3E). Chloroflexi were found at relatively high abundance in the hot spring source sample of 64 °C (TP64 = 12.1%), ETP = 31% (Fig. 3F). The archaeal phylum Korarchaeota was observed in all ETP samples but not in the TP samples (Fig. S1). The other three archaea phyla, Crenarchaeota, Euryarchaeota, and Thaumarchaeota, were found in all samples of both groups at <1.0% relative abundance, except Euryarchaeota, which was detected at 4.7%, 1.9%, and 1.0% in ETP60, ETP55, and TP64, respectively.

### Families analysis

Diversity between enriched and non-enriched samples were visible at the family level. We observed 259 bacterial families in the TP samples, 283 in the ETP samples, and only 123 that were common to all samples. From hot spring source sample, we identified 203 bacterial families in TP64, of which 168 also were retrieved from ETP64. Among archaea, 22 families were identified in the TP samples and 26 in the ETP samples. Among OTUs, 13 families were common to both TP64 and ETP64. Table S4 summarizes the differences in common families between the different samples of both groups.

**Figure 4:**
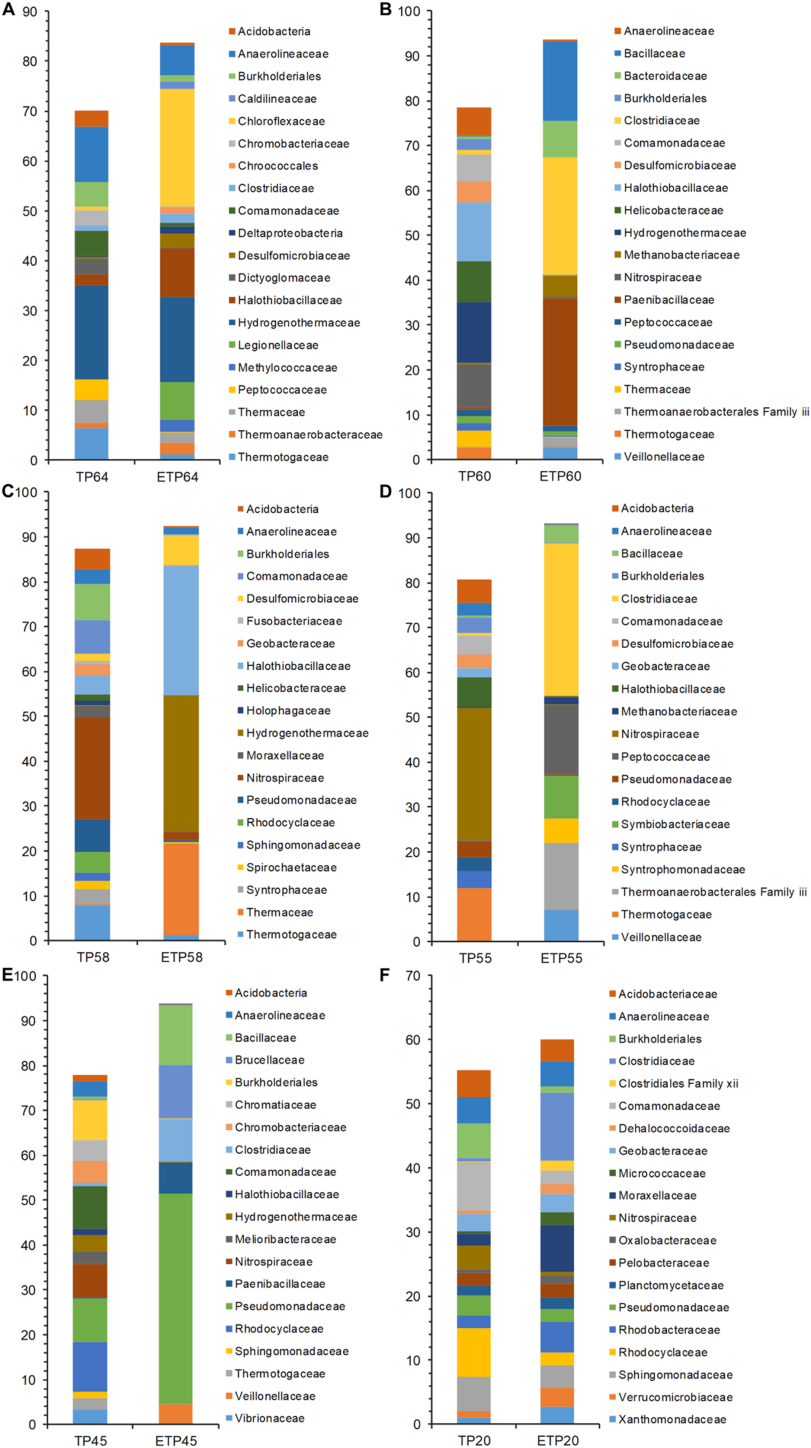
Comparative analysis of 20 most abundant families in TP and ETP sample of different temperature. The bar chart represents the relative abundance of families in percentages between TP and ETP samples collected at temperature gradients (A) 64 °C, (B) 60 °C, (C) 58 °C, (D) 55 °C, (E) 45 °C, and (F) 20 °C. The alphabetical letters in sample name represent sample group and numeric value indicate temperature in centigrade. TP, sediment samples from Tatta Pani hot spring; ETP, enriched sediment samples from Tatta Pani hot spring.

The most dominant families in the TP groups included Hydrogenothermaceae from the high-temperature TP64 (18.8%) and TP60 (13.5%) samples (Figs. 4A–4F), Nitrospiraceae from the TP58 (22.7%) and TP55 (29.5%) samples, and Rhodocyclaceae from the low-temperature TP45 (11.1%) and TP20 (7.6%) samples. Abundant families in the enrichment samples included Chloroflexaceae (23.7%) and Hydrogenothermaceae (16.9%) from ETP64. We also observed more variation in abundance of dominant families in other enrichment samples, compared with their respective non-enriched samples (Figs. 4A–4F). For example, Hydrogenothermaceae was abundant in ETP58 (30.2%) than TP58 (0.1%), and Clostridiaceae showed more abundance (≥9%) in the ETP60, ETP55, ETP45, and ETP20 samples than in any TP sample (≤1%) (Figs. 4A–4F).

### Genera comparative analysis

**Figure 5:**
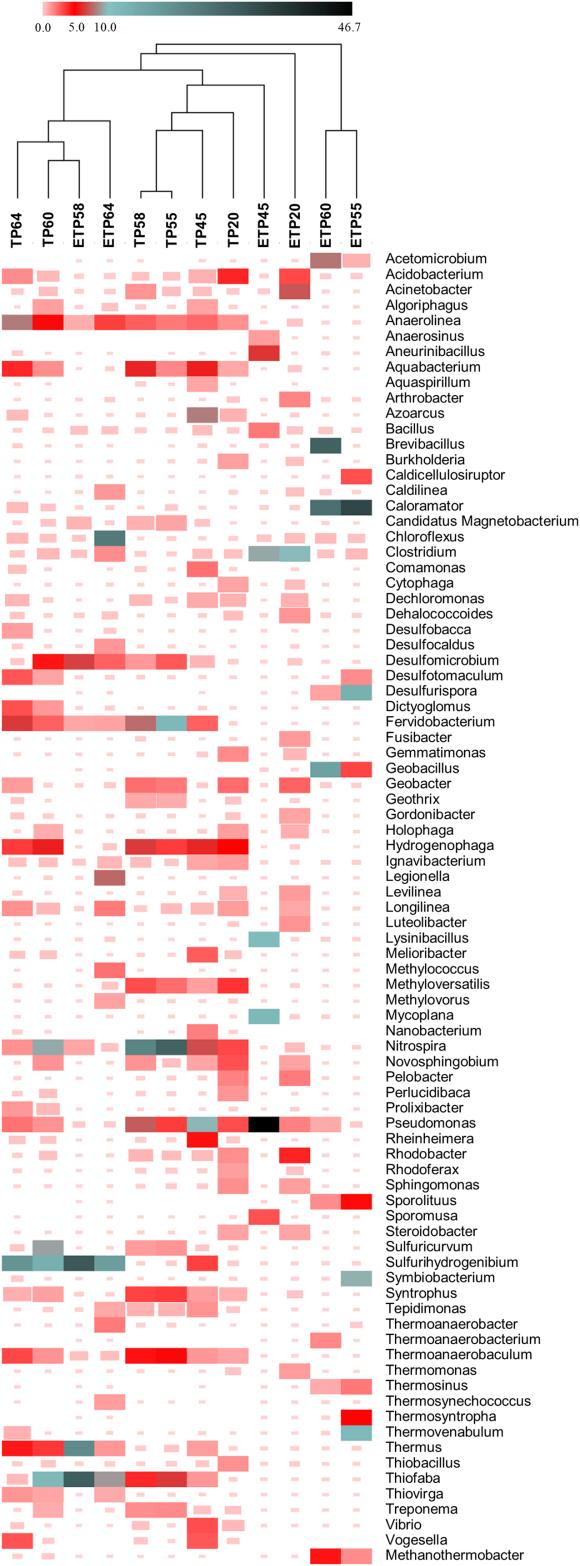
Heatmap showing percentage relative abundance (≥1%) of genera in at least one enriched or non-enriched sample from Tatta Pani hot spring. Sample clustering was performed based on the Pearson correlation. The alphabetical letters in the sample name represent the sample group, and the numeric value indicates the temperature in centigrade. TP, sediment samples from Tatta Pani hot spring; ETP, enriched sediment samples from Tatta Pani hot spring.

In the TP samples, we identified 725 bacteria and 31 archaeal genera (Table S5). Among them, 241 bacteria and 8 archaeal genera were common across samples from different temperatures of TP, and 15 bacterial genera were unique to hot spring source sample TP64 of temperature 64 °C. Genera of *Nitrospira* (11.9 ± 10.7%), *Pseudomonas* (4.6 ± 3.2%), *Hydrogenophaga* (5.1 ± 1.2%), and *Anaerolinea* (3.8 ± 2.5%) were prevalent at all temperatures (Fig. 5). Thermophilic genera *Sulfurihydrogenibium*, *Fervidobacterium*, *Thermus*, *Thermoanaerobaculum*, *Dictyoglomus*, and *Desulfotomaculum* were dominant in the ≥60 °C samples. *Nitrospira* was relatively more predominant (>21%) in the 58 °C and 55 °C samples (Fig. 5). Mesophilic genera *Pseudomonas* (9.7%) and *Azoarcus* (8.2%) were observed in TP45, and *Acidobacterium* (4.2%) was observed in TP20. Overall, the relative abundance of thermopiles in the TP samples decreased as the respective temperature decreased.

In the ETP samples, 609 bacterial genera were retrieved from 725 genera found in the TP samples (Table S5). Moreover, 273 genera were commonly found in ETP64 from the 454 genera detected in TP64, and the ETP20 and TP20 samples shared 505 genera (Table S5). Similarly, ≥250 genera were common to both groups at the other respective temperatures. Further, analysis of the rare genera detected at <0.01% relative abundance revealed that 77 genera were commonly found in ≥50% samples from TP and ETP groups. Moreover, increased number of rare genera were retrieved specifically in the ETP samples that were not detected in TP samples except TP20 carrying an increased number of rare genera uniquely than ETP20 (Fig. S3).

Substantial variations were observed in the relative abundance of genera between the enriched and non-enriched samples at each temperature. For example, *Chloroflexus* was predominantly found in ETP64 (23.7%) but not in the other samples (<1%). *Brevibacillus*, *Caloramator*, and *Geobacillus* were dominantly found in ETP60 (>15%) but not in the TP samples (<1%). *Caloramator* was observed in ETP55 (33%). *Sulfurihydrogenibium*, *Thiofaba*, and *Thermus* were abundant (>20%) in ETP58. *Pseudomonas* was particularly abundant (46.7%) at 45 °C, whereas the pathogenic genera *Clostridium* (9.8%) and *Acinetobacter* (7.2%) were most abundant in the 20 °C ETP20 sample. A considerable difference was observed in relative abundance of the sulfur and nitrogen metabolism-related genera between TP and ETP samples. Total, 79 genera were detected carrying known sulfur metabolism-associated bacteria in the Tatta Pani hot spring. Among them, 33 genera were commonly found in more than 50% of both groups’ samples. However, a relative abundance of the dominant genera, *Fervidobacterium* (6.5%), *Thermoanaerobaculum* (3.3%), and *Desulfotomaculum* (3.0%) detected in the hot spring source sample TP64 was decreased to ≤1.0% in ETP64 sample. Majority of the sulfur metabolizing genera were found in <0.01% relative abundance in both TP and ETP samples. Collectively, 27 rare genera were uniquely found in ETP samples compared to six genera were found specifically in TP samples. Among 34 genera carrying known nitrogen metabolism associated bacteria, eight genera were uniquely found in ETP samples. Genus *Nitrospira* was found at a higher abundance of 11.9% in hot spring source sample TP64 and was substantially decreased to 0.4% in ETP64 sample.

### Enrichment-induced changes in diversity and community composition

Considerable differences in diversity and relative abundance were observed between the ETP and TP groups at all taxonomic levels. Two rare bacterial phyla of relative abundance (<0.01%), Candidatus Cloacimonetes and Caldiserica, were unique to the ETP group. The relative abundance of phylum Chlorobi was increased in ETP samples that was detected in <0.01% abundance in most of the TP samples. Phylum Thermodesulfobacteria identified only in the non-enriched TP64 was commonly retrieved with enrichment in ETP samples (<0.01%). The rare bacterial phylum Nitrospinae was commonly found in ≥50% of the TP and ETP samples. Moreover, the archaeal phylum Korarchaeota was common in the ETP samples at relative abundance of <0.01% but not detected in the TP samples (Fig. S1). Furthermore, 1,192 and 1,648 totals bacterial OTUs were retrieved from the TP and ETP groups, respectively, with 97% similarity (Table S6), indicating that enrichment increased species diversity. The cumulative abundance of unique OTU diversity in the ETP samples (*n* = 805, 69.8%) exceeded that of the TP samples (*n* = 349, 30.2%). Most unique OTUs came from Proteobacteria, followed by Firmicutes, in both groups.

We also analyzed OTUs that were retrieved or lost with enrichment. In ETP64, 302 unique OTUs were retrieved, compared to 359 in TP64. In ETP20, 691 unique OTUs were retrieved, compared to 263 unique OTUs with its parent TP20 sample. From archaea, 80 OTUs were found in the ETP group samples, compared with 44 from the TP group samples. Increased diversity of OTUs was found in the ≤60 °C ETP samples. Out of 29 OTUs from TP64, 16 were retrieved from ETP64. Table S6 summarizes the results.

### Culture-dependent analysis and comparison with 16S amplicon sequencing based diversity

We isolated 145 strains and identified 56 species using MALDI-TOF and 16S rRNA sequencing of the TP64 sediment sample (Fig. S4A). Among them, 6.8% of isolates grew at 60 °C. Proteobacteria (*n* = 74, 51%) were most common, followed by Firmicutes (*n* = 59, 40.6%) and Bacteroidetes (*n* = 10, 6.89%) (Fig. S4B). Compared with 203 families identified from the 16S amplicon sequencing data of TP64, 17 were identified in the culture-dependent analysis, mainly Bacillaceae (*n* = 46, 31.72%), Pseudomonadaceae (*n* = 45, 31%), and Caulobacteraceae (*n* = 15, 10.34%) (Fig. S4C). The most dominant species were *Bacillus cereus*, *Pseudomonas grimontii*, *Pseudomonas veronii*, *Pseudomonas fragi*, *Brevundimonas vesicularis*, *Bacillus pumilus*, *Paenisporosarcina quisquiliarum*, and *Flavobacterium hibernum* (Fig. S4A). The isolate MY-TP24 showed 95.8% similarity with *Chelatococcus reniformis*. A distinct clade in the 16S rRNA gene phylogenetic tree, identified from closely related type strains, indicates a potential novel candidate species (Fig. S5).

**Figure 6:**
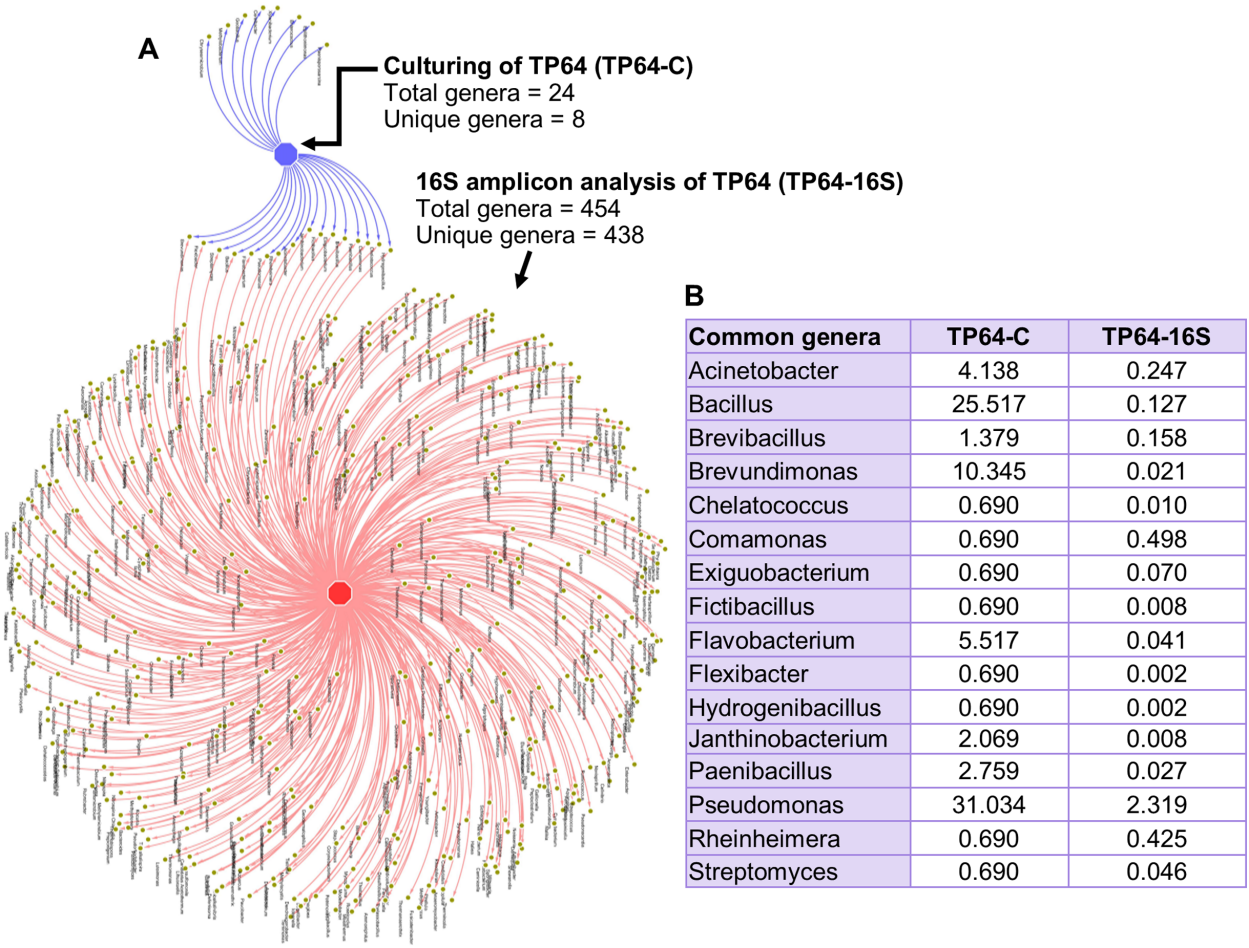
Analysis of common and unique genera identified from culture-dependent analysis of the Tatta Pani hot spring sample TP64 (TP64-C), compared with 16S amplicon sequence analysis of non-enriched TP64 sample (TP64-16S). (A) The circular nodes in the network attached with two edges indicate common genera between TP64-C and TP64-16S. (B) The percentage of relative abundance of the common genera between TP64-C and TP64-16S.

The percentage abundance of Firmicutes and Bacteroidetes in TP64 was much higher in the culture-dependent analysis than in the 16S amplicon analysis (40.6% vs. 9.6% and 6.9% vs. 1.7%, respectively). Proteobacteria were dominant in both culture-dependent (51%) and 16S amplicon (30%) analyses. Actinobacteria were found at relatively lower abundances in both culture-dependent (1.4%) and 16S amplicon (1.2%) analyses. Out of 24 cultured genera, 16S amplicon analysis detected 16 (Fig. 6).

## Discussion

Among geothermal systems around the world, hot springs are easily accessible and comprise specific microbial communities acclimatized to stringent environments (Kumar, 2014). Yet, few traditional culture-dependent studies and only one 16S amplicon study have been conducted on the Tatta Pani hot spring in Azad Kashmir (Jadoon et al., 2014; Javed et al., 2012; Zahoor, Javed & Aftab, 2012; Amin et al., 2017). The water flow of these hot springs varies from 4.3 to 11.8 L/s (Anees, Shah & Qureshi, 2015; Zaigham, 2005; Zaigham, Nayyar & Hisamuddin, 2009). Tatta Pani hot spring is neutral to alkaline in nature, and containing sulfate of 410–460 mg/L reported previously (Amin et al., 2017; Anees, Shah & Qureshi, 2015; Zaigham, 2005). In this study, multivariate principal coordinate and cluster analysis revealed a major change in microbial diversity between TP and ETP samples. The PCoA plot shows that samples from TP of close temperature cluster together in agreement with previous studies reported that temperature is an important determinant of shaping microbial diversity and leads to distinct microbial communities according to temperature gradient (Chan et al., 2017; Inskeep et al., 2013; Ward et al., 2017). However, the microbial diversity increase with temperature decrease was not linear in this study, as observed in some of the previous studies (Ghilamicael et al., 2017; Podar et al., 2020; Zhang et al., 2018). The disconnection in clustering patterns between TP and ETP samples of the same temperature indicates that although temperature is an important factor, it is not an exclusive determinant of microbial diversity. Several studies documented the role of other environmental and physicochemical factors in shaping the microbial communities; for example, changes in water chemistry, minerals, and organic substrates may be driving the specific changes that we did not measure (Chan et al., 2017; Colman et al., 2019; Inskeep et al., 2013). In agreement with our finding, Zhang et al. (2018) identified that abundant, intermediate, and rare microbial communities responded differently to temperature and suggested that various microbial taxa distribution in hot springs are selectively affected by different environmental factors.

Tatta Pani hot spring has abundant Proteobacteria, of which most OTUs belong to the family Comamonadaceae. Previously, remarkable phenotypic diversity observed in taxa from Comamonadaceae that includes fermentative, hydrogen oxidizing, anaerobic denitrifying, aerobic organotrophic, and photoautotrophic bacteria (Willems, 2014). The hydrogen-oxidizing genus *Hydrogenphaga* from the family Comamonadaceae, previously reported in hot springs in Japan (Hayashi et al., 1999) and Yellowstone National Park in Wyoming, USA (Stöhr et al., 2001). The ecologically important sulfur-oxidizing species *Thiofaba tepidiphila* from γ-Proteobacteria, which was first identified in Akayu sulfur hot spring in Fukushima was predominantly found in Tatta Pani hot springs (Mori & Suzuki, 2008). This bacterium plays a role in the biogeochemical cycling of the inhabiting ecosystem’s sulfur (Mori & Suzuki, 2008). Consistently, Amin et al. reported Proteobacteria as the most abundant phylum from Tatta Pani hot spring, with OTUs belonging to genera *Methyligella*, *Desulfatirhabdium, Desulfomicrobium*, and the halotolerant class Dehalococcoidetes of *Chloroflexi* (Amin et al., 2017).

Moreover, we found the extremophilic phylum *Aquificae* in abundance (>16%) in the 64 °C samples, which were not detected in the previous 16S amplicon-based analyses from this site (Amin et al., 2017). Taxa belonging to this phylum have adapted to the harsh hot spring environment. For example, the sulfur-oxidizing, chemolithoautotrophic, and thermophilic species of genus *Sulfurihydrogenibium* from the family *Hydrogentheramcea* was discovered in hot springs of Yellowstone National Park, and Azores (Aguiar, Beveridge & Reysenbach, 2004; Nakagawa et al., 2005). The genus *Sulfurihydrogenibium* also was present in high abundance in our TP samples. The 64 °C TP samples were rich in genera belonging exclusively to the thermophilic families *Thermaceae* (obligately oxidative, phyla Deinococcus-Thermus) and *Dictyoglomaceae* (phyla Dictyoglomi) (Albuquerque & Da Costa, 2014; Bergquist & Morgan, 2014), along with taxa from hyperthermophilic and thermophilic phyla *Aquificae* and *Thermotogae*. In consistent with previous studies, Tatta Pani hot spring also has relative high abundance of OTUs belonging to the genus *Fervidobacterium* from phylum *Thermotogae* (Kanoksilapatham et al., 2016; Wang et al., 2010). Furthermore, the TP64 sample was exclusively rich in the thermophilic methanotroph *Methylothermus*, a specialized group of bacteria that uses methane and methanol as sole carbon and energy sources (Tsubota et al., 2005).

Our study revealed that enrichment of hot spring sediment increased bacterial diversity and induced growth of rare taxa that were not detected in the original samples. However, enrichment also decreased dominance of thermophilic taxa, which were prominent in the original samples. For instance, dominance of thermophilic phyla *Thermotogae*, *Dictyoglomi*, *Nitrospirae*, *Ignavibacteriae*, *Acidobacteria* markedly decreased in the ETP samples. Moreover, the relatively dominant microbiota varied between the enriched and non-enriched groups. For example, Chloroflexi increased to 31.7% in ETP64, compared with 11.9% in its non-enriched counterpart. Similarly, the thermophilic sulfate-reducing bacteria *Thermodesulfobacteria* found in ETP64 was not detected in the TP64 sample (Jeanthon et al., 2002). The thermophilic, anoxygenic, phototrophic, green, non-sulfur bacteria genus of *Chloroflexus* belonging to *Chloroflexaceae* was most dominant in ETP64, and it has been identified in hot springs around the world (Amin et al., 2017; Gaisin et al., 2015; Tank et al., 2017). *Thermoanaerobacter* from phylum Firmicutes also was uniquely abundant in the ETP groups. The anaerobic, sulfur-producing species from this genus was initially found in a hot spring of New Zealand (Lee et al., 2007). However, ETP64 also exhibited decreased dominance in thermophilic core microbiota, compared with the TP64 sample. The decrease of thermophilic bacteria in the ETP samples is probably because of lacking in the laboratory condition of the geothermal habitat interactive environment of specific sediment nutrient composition and environmental factors other than temperature that might require for the growth of those particular thermophilic bacteria. Even though we used the low nutrient media considering the hot springs’ general oligotrophic nature following methodology previously reported to maximize the culture and uncultured bacteria from environmental samples (Vartoukian, Palmer & Wade, 2010; Yasir et al., 2019). The media was supplemented with 50% of the sterilized hot spring water to support the inhabitant microbiota’s specific growth requirement. Studies report varying physicochemical properties of Tatta Pani hot springs, with temperatures ranging from 42 °C to 83 °C and pH ranging from 7.0 to 8.8 (Ahmad et al., 2002; Amin et al., 2017; Anees, Shah & Qureshi, 2015; Zahoor, Javed & Aftab, 2012). Recent studies have recorded temperatures between 59.2 °C and 60.7 °C and pH between 6.2 and 9.4 (Amin et al., 2017; Anees, Shah & Qureshi, 2015). Moreover, differences in microbial dominance have been observed between enriched and non-enriched temperature-matched samples. Our finding of high numbers of sulfur- and nitrogen-metabolizing bacteria correlate with high amounts of sulfur and nitrogen found in Tatta Pani hot springs (Amin et al., 2017; Anees, Shah & Qureshi, 2015).

Microbial culture methods are vital in developing whole-cell applications and understanding the biochemical (e.g., thermostable enzyme production) and physiological nature of pure isolates. Culture-dependent analysis of sediment from Tatta Pani hot spring resulted in isolation and identification of species belonging mostly to Proteobacteria, followed by Firmicutes, Bacteroidetes, and Actinobacteria. In consistent with previous studies (Yasir et al., 2019), we also found that species from the Bacillus genus were common. Bacterial taxa retrieved in the culture-dependent method were not completely identified in 16S amplicon sequencing data. It is consistent with previous studies that showed partial overlapping in coverage of bacterial communities between the two methods (Amrane et al., 2019; Khan et al., 2019; Lagier et al., 2012). Probably, 16S amplicon sequencing might miss the low abundant bacteria, and the specific culture media support the growth of low abundant species as previously noticed (Diakite et al., 2019; Khan et al., 2019). We isolated a MY-TP24 strain as a potential novel candidate species showing close branching with uncultured bacteria. Previously, Amin et al. reported a novel species from the Tatta Pani hot springs (Amin et al., 2016). Together, these findings suggest unexplored diversity of novel bacteria in the Tatta Pani hot spring.

## Conclusions

We identified high thermophilic bacterial diversity in samples collected from non-volcanic Tatta Pani hot spring. Comparative analyses of enriched and non-enriched samples provided insight about rare taxa, revealing differing compositions of dominant bacteria, along with variation in the abundance of thermophilic bacteria. Culture-dependent analysis exhibiting potential for possible isolation of novel species. In conclusion, enrichment enhanced the retrieval of rare taxa but changed the overall microbial community and should be avoided to describe the hot spring microbiota composition. The culture-independent method provided a comprehensive analysis of the microbial community in hot springs. Further studies are recommended to improve culture-dependent methods for the isolation of hot spring microbiota.

## Supplemental Information

10.7717/peerj.10995/supp-1 Supplemental Information 1 Supplemental figures and tables.

## Additional Information and Declarations

### Field Study Permissions

The following information was supplied relating to field study approvals (i.e., approving body and any reference numbers): No specific permission was required for sampling the studied hot spring that was not privately owned or protected in any way. The hot spring was not part of a national park or reserve.

### Data Availability

The following information was supplied regarding data availability: Date are available from the European Nucleotide Archive: SAMEA6812218–SAMEA6812229. Data are also available at ENA: PRJEB38122.

## References

[ref-1] Aguiar P, Beveridge TJ, Reysenbach AL (2004). Sulfurihydrogenibium azorense, sp. nov., a thermophilic hydrogen-oxidizing microaerophile from terrestrial hot springs in the Azores. International Journal of Systematic and Evolutionary Microbiology.

[ref-2] Ahmad M, Akram W, Ahmad N, Tasneem MA, Rafiq M, Latif Z (2002). Assessment of reservoir temperatures of thermal springs of the northern areas of Pakistan by chemical and isotope geothermometry. Geothermics.

[ref-3] Albuquerque L, Da Costa MS, DeLong EF, Lory S, Stackebrandt E, Thompson F (2014). The family thermaceae. The Prokaryotes.

[ref-4] Amin A, Ahmed I, Habib N, Abbas S, Xiao M, Hozzein WN, Li W-J (2016). Nocardioides pakistanensis sp. nov., isolated from a hot water spring of Tatta Pani in Pakistan. Antonie van Leeuwenhoek.

[ref-5] Amin A, Ahmed I, Salam N, Kim B-Y, Singh D, Zhi X-Y, Xiao M, Li W-J (2017). Diversity and distribution of thermophilic bacteria in hot springs of Pakistan. Microbial Ecology.

[ref-6] Amrane S, Hocquart M, Afouda P, Kuete E, Pham TP, Dione N, Ngom II, Valles C, Bachar D, Raoult D, Lagier JC (2019). Metagenomic and culturomic analysis of gut microbiota dysbiosis during Clostridium difficile infection. Scientific Reports.

[ref-7] Anees M, Shah MM, Qureshi AA (2015). Isotope studies and chemical investigations of tattapani hot springs in Kotli (Kashmir, NE Pakistan): implications on reservoir origin and temperature. Procedia Earth and Planetary Science.

[ref-8] Angelakis E, Yasir M, Azhar EI, Papadioti A, Bibi F, Aburizaiza AS, Metidji S, Memish ZA, Ashshi AM, Hassan AM, Harakeh S, Gautret P, Raoult D (2014). MALDI-TOF mass spectrometry and identification of new bacteria species in air samples from Makkah, Saudi Arabia. BMC Research Notes.

[ref-9] Baioumy H, Nawawi M, Wagner K, Arifin MH (2015). Geochemistry and geothermometry of non-volcanic hot springs in West Malaysia. Journal of Volcanology Geothermal Research.

[ref-10] Bakht MS (2000). An overview of geothermal resources of Pakistan.

[ref-11] Barns SM, Fundyga RE, Jeffries MW, Pace NR (1994). Remarkable archaeal diversity detected in a Yellowstone National Park hot spring environment. Proceedings of the National Academy of Sciences of the United States of America.

[ref-12] Bergquist P, Morgan H, DeLong EF, Lory S, Stackebrandt E, Thompson F (2014). The phylum dictyoglomi. The Prokaryotes.

[ref-13] Boyd ES, Hamilton TL, Spear JR, Lavin M, Peters JW (2010). [FeFe]-hydrogenase in Yellowstone National Park: evidence for dispersal limitation and phylogenetic niche conservatism. ISME Journal.

[ref-14] Caporaso JG, Kuczynski J, Stombaugh J, Bittinger K, Bushman FD, Costello EK, Fierer N, Pena AG, Goodrich JK, Gordon JI, Huttley GA, Kelley ST, Knights D, Koenig JE, Ley RE, Lozupone CA, McDonald D, Muegge BD, Pirrung M, Reeder J, Sevinsky JR, Turnbaugh PJ, Walters WA, Widmann J, Yatsunenko T, Zaneveld J, Knight R (2010). QIIME allows analysis of high-throughput community sequencing data. Nature Methods.

[ref-15] Chan CS, Chan KG, Ee R, Hong KW, Urbieta MS, Donati ER, Shamsir MS, Goh KM (2017). Effects of physiochemical factors on prokaryotic biodiversity in Malaysian circumneutral hot springs. Frontiers in Microbiology.

[ref-16] Colman DR, Lindsay MR, Amenabar MJ, Boyd ES (2019). The intersection of geology, geochemistry, and microbiology in continental hydrothermal systems. Astrobiology.

[ref-17] Cousins CR, Fogel M, Bowden R, Crawford I, Boyce A, Cockell C, Gunn M (2018). Biogeochemical probing of microbial communities in a basalt-hosted hot spring at Kverkfjoll volcano. Iceland Geobiology.

[ref-18] Daniel R (2013). Microbial diversity and biochemical potential encoded by thermal spring metagenomes derived from the Kamchatka Peninsula. Archea.

[ref-19] Diakite A, Dubourg G, Dione N, Afouda P, Bellali S, Ngom II, Valles C, Million M, Levasseur A, Cadoret F, Lagier JC, Raoult D (2019). Extensive culturomics of 8 healthy samples enhances metagenomics efficiency. PLOS ONE.

[ref-20] Gaisin VA, Kalashnikov AM, Sukhacheva MV, Namsaraev ZB, Barhutova DD, Gorlenko VM, Kuznetsov BB (2015). Filamentous anoxygenic phototrophic bacteria from cyanobacterial mats of Alla hot springs (Barguzin Valley, Russia). Extremophiles.

[ref-21] Ghilamicael AM, Budambula NLM, Anami SE, Mehari T, Boga HI (2017). Evaluation of prokaryotic diversity of five hot springs in Eritrea. BMC Microbiology.

[ref-22] Hayashi NR, Ishida T, Yokota A, Kodama T, Igarashi Y (1999). Hydrogenophilus thermoluteolus gen. nov., sp. nov., a thermophilic, facultatively chemolithoautotrophic, hydrogen-oxidizing bacterium. International Journal of Systematic and Evolutionary Microbiology.

[ref-23] Inskeep W, Jay Z, Tringe S, Herrgard M, Rusch D (2013). The YNP metagenome project: environmental parameters responsible for microbial distribution in the yellowstone geothermal ecosystem. Frontiers in Microbiology.

[ref-24] Inskeep WP, Rusch DB, Jay ZJ, Herrgard MJ, Kozubal MA, Richardson TH, Macur RE, Hamamura N, De Jennings RM, Fouke BW (2010). Metagenomes from high-temperature chemotrophic systems reveal geochemical controls on microbial community structure and function. PLOS ONE.

[ref-25] Jadoon MA, Ahmad T, Rehman MMU, Khan A, Majid A (2014). Isolation and identification of thermophillic actinomycetes from hot water springs from azad Jammu and Kashmir Pakistan for the production of thermophillic amylase. World Applied Sciences Journal.

[ref-26] Javed MM, Zahoor S, Sabar H, Haq IU, Babar ME (2012). Thermophilic bacteria from the hot springs of Gilgit (Pakistan). Journal of Animal and Plant Sciences.

[ref-27] Jeanthon C, L’Haridon S, Cueff V, Banta A, Reysenbach A-L, Prieur D (2002). Thermodesulfobacterium hydrogeniphilum sp. nov., a thermophilic, chemolithoautotrophic, sulfate-reducing bacterium isolated from a deep-sea hydrothermal vent at Guaymas Basin, and emendation of the genus Thermodesulfobacterium. International Journal of Systematic Evolutionary Microbiology.

[ref-28] Jiang X, Takacs-Vesbach CD (2017). Microbial community analysis of pH 4 thermal springs in Yellowstone National Park. Extremophiles.

[ref-29] Jousset A, Bienhold C, Chatzinotas A, Gallien L, Gobet A, Kurm V, Küsel K, Rillig MC, Rivett DW, Salles JF, Van der Heijden MGA, Youssef NH, Zhang X, Wei Z, Hol WHG (2017). Where less may be more: how the rare biosphere pulls ecosystems strings. ISME Journal.

[ref-30] Kanoksilapatham W, Pasomsup P, Keawram P, Cuecas A, Portillo MC, Gonzalez JM (2016). Fervidobacterium thailandense sp. nov., an extremely thermophilic bacterium isolated from a hot spring. International Journal of Systematic Evolutionary Microbiology.

[ref-31] Khan I, Yasir M, Farman M, Kumosani T, AlBasri SF, Bajouh OS, Azhar EI (2019). Evaluation of gut bacterial community composition and antimicrobial resistome in pregnant and non-pregnant women from Saudi population. Infection and Drug Resistance.

[ref-32] Kim O-S, Cho Y-J, Lee K, Yoon S-H, Kim M, Na H, Park S-C, Jeon YS, Lee J-H, Yi H, Won S, Chun J (2012). Introducing EzTaxon-e: a prokaryotic 16S rRNA gene sequence database with phylotypes that represent uncultured species. International Journal of Systematic and Evolutionary Microbiology.

[ref-33] Kumar M (2014). Deciphering the diversity of culturable thermotolerant bacteria from Manikaran hot springs. Annals of Microbiology.

[ref-34] Kumar S, Stecher G, Li M, Knyaz C, Tamura K (2018). MEGA X: molecular evolutionary genetics analysis across computing platforms. Molecular Biology and Evolution.

[ref-35] Lagier JC, Armougom F, Million M, Hugon P, Pagnier I, Robert C, Bittar F, Fournous G, Gimenez G, Maraninchi M, Trape JF, Koonin EV, La Scola B, Raoult D (2012). Microbial culturomics: paradigm shift in the human gut microbiome study. Clinical Microbiology and Infection.

[ref-36] Lee Y-J, Dashti M, Prange A, Rainey FA, Rohde M, Whitman WB, Wiegel J (2007). Thermoanaerobacter sulfurigignens sp. nov., an anaerobic thermophilic bacterium that reduces 1 M thiosulfate to elemental sulfur and tolerates 90 mM sulfite. International Journal of Systematic and Evolutionary Microbiology.

[ref-37] Masella AP, Bartram AK, Truszkowski JM, Brown DG, Neufeld JD (2012). PANDAseq: paired-end assembler for illumina sequences. BMC Bioinformatics.

[ref-38] Miller SR, Strong AL, Jones KL, Ungerer MC (2009). Bar-coded pyrosequencing reveals shared bacterial community properties along the temperature gradients of two alkaline hot springs in Yellowstone National Park. Applied and Environmental Microbiology.

[ref-39] Mori K, Suzuki K-I (2008). Thiofaba tepidiphila gen. nov., sp. nov., a novel obligately chemolithoautotrophic, sulfur-oxidizing bacterium of the Gammaproteobacteria isolated from a hot spring. International Journal of Systematic Evolutionary Microbiology.

[ref-40] Nakagawa S, Shtaih Z, Banta A, Beveridge TJ, Sako Y, Reysenbach AL (2005). Sulfurihydrogenibium yellowstonense sp. nov., an extremely thermophilic, facultatively heterotrophic, sulfur-oxidizing bacterium from Yellowstone National Park, and emended descriptions of the genus Sulfurihydrogenibium, Sulfurihydrogenibium subterraneum and Sulfurihydrogenibium azorense. International Journal of Systematic and Evolutionary Microbiology.

[ref-41] Payne D, Dunham EC, Mohr E, Miller I, Arnold A, Erickson R, Fones EM, Lindsay MR, Colman DR, Boyd ES (2019). Geologic legacy spanning >90 years explains unique Yellowstone hot spring geochemistry and biodiversity. Environmental Microbiology.

[ref-42] Podar PT, Yang Z, Bjornsdottir SH, Podar M (2020). Comparative analysis of microbial diversity across temperature gradients in hot springs from yellowstone and Iceland. Frontiers in Microbiology.

[ref-43] Poddar A, Das SK (2018). Microbiological studies of hot springs in India: a review. Archives of Microbiology.

[ref-44] Power JF, Carere CR, Lee CK, Wakerley GLJ, Evans DW, Button M, White D, Climo MD, Hinze AM, Morgan XC, McDonald IR, Cary SC, Stott MB (2018). Microbial biogeography of 925 geothermal springs in New Zealand. Nature Communications.

[ref-45] Sakai HD, Kurosawa N (2016). Exploration and isolation of novel thermophiles in frozen enrichment cultures derived from a terrestrial acidic hot spring. Extremophiles.

[ref-46] Schröder C, Elleuche S, Blank S, Antranikian G (2014). Characterization of a heat-active archaeal β-glucosidase from a hydrothermal spring metagenome. Enzyme Microbial Technology.

[ref-47] Shah AA, Nawaz A, Kanwal L, Hasan F, Khan S, Badshah M (2015). Degradation of poly(ε-caprolactone) by a thermophilic bacterium Ralstonia sp. strain MRL-TL isolated from hot spring. International Biodeterioration Biodegradation.

[ref-48] Sheikh MA (2009). Renewable energy resource potential in Pakistan. Renewable and Sustainable Energy Reviews.

[ref-49] Stöhr R, Waberski A, Liesack W, Völker H, Wehmeyer U, Thomm M (2001). Hydrogenophilus hirschii sp. nov., a novel thermophilic hydrogen-oxidizing beta-proteobacterium isolated from Yellowstone National Park. International Journal of Systematic and Evolutionary Microbiology.

[ref-50] Swingley WD, D’Arcy R, Shock EL, Alsop EB, Falenski HD, Havig JR, Raymond J (2012). Coordinating environmental genomics and geochemistry reveals metabolic transitions in a hot spring ecosystem. PLOS ONE.

[ref-51] Tank M, Thiel V, Ward DM, Bryant DA (2017). A panoply of phototrophs: an overview of the thermophilic chlorophototrophs of the microbial mats of alkaline siliceous hot springs in Yellowstone National Park, WY, USA.

[ref-52] Tindall BJ, Rossello-Mora R, Busse HJ, Ludwig W, Kampfer P (2010). Notes on the characterization of prokaryote strains for taxonomic purposes. International Journal of Systematic and Evolutionary Microbiology.

[ref-53] Tsubota J, Eshinimaev BT, Khmelenina VN, Trotsenko YA (2005). Methylothermus thermalis gen. nov., sp. nov., a novel moderately thermophilic obligate methanotroph from a hot spring in Japan. International Journal of Systematic and Evolutionary Microbiology.

[ref-54] Vartoukian SR, Palmer RM, Wade WG (2010). Strategies for culture of ‘unculturable’ bacteria. FEMS Microbiology Letters.

[ref-55] Verma A, Dhiman K, Gupta M, Shirkot P (2015). Bioprospecting of thermotolerant bacteria from Hot Water Springs of Himachal Pradesh for the production of Taq DNA polymerase. Proceedings of the National Academy of Sciences, India Section B: Biological Sciences.

[ref-56] Wang Q, Cen Z, Zhao J (2015). The survival mechanisms of thermophiles at high temperatures: an angle of omics. Physiology.

[ref-57] Wang Y, Wang X, Tang R, Yu S, Zheng B, Feng Y (2010). A novel thermostable cellulase from Fervidobacterium nodosum. Journal of Molecular Catalysis B: Enzymatic.

[ref-58] Ward L, Taylor MW, Power JF, Scott BJ, McDonald IR, Stott MB (2017). Microbial community dynamics in Inferno Crater Lake, a thermally fluctuating geothermal spring. ISME Journal.

[ref-59] Wilkins LGE, Ettinger CL, Jospin G, Eisen JA (2019). Metagenome-assembled genomes provide new insight into the microbial diversity of two thermal pools in Kamchatka. Scientific Reports.

[ref-60] Willems A, Rosenberg E, DeLong EF, Lory S, Stackebrandt E, Thompson F (2014). The family comamonadaceae. The Prokaryotes: Alphaproteobacteria and Betaproteobacteria.

[ref-61] Yamamoto K, Hackley KC, Kelly WR, Panno SV, Sekiguchi Y, Sanford RA, Liu WT, Kamagata Y, Tamaki H (2019). Diversity and geochemical community assembly processes of the living rare biosphere in a sand-and-gravel aquifer ecosystem in the Midwestern United States. Scientific Reports.

[ref-62] Yasir M, Angelakis E, Bibi F, Azhar EI, Bachar D, Lagier JC, Gaborit B, Hassan AM, Jiman-Fatani AA, Alshali KZ, Robert C, Dutour A, Raoult D (2015). Comparison of the gut microbiota of people in France and Saudi Arabia. Nutrition & Diabetes.

[ref-63] Yasir M, Aslam Z, Kim SW, Lee SW, Jeon CO, Chung YR (2009). Bacterial community composition and chitinase gene diversity of vermicompost with antifungal activity. Bioresource Technology.

[ref-64] Yasir M, Qureshi AK, Khan I, Bibi F, Rehan M, Khan SB, Azhar EI (2019). Culturomics-based taxonomic diversity of bacterial communities in the hot springs of Saudi Arabia. OMICS: A Journal of Integrative Biology.

[ref-65] Zahoor S, Javed MM, Aftab MN (2012). Isolation and molecular identification of a facultatively anaerobic bacterium from the hot spring of Azad Kashmir. Pakistan Journal of Botany.

[ref-66] Zaigham NA (2005). Geothermal energy resources of Pakistan.

[ref-67] Zaigham NA, Nayyar ZA, Hisamuddin N (2009). Review of geothermal energy resources in Pakistan. Renewable Sustainable Energy Reviews.

[ref-68] Zhang Y, Wu G, Jiang H, Yang J, She W, Khan I, Li W (2018). Abundant and rare microbial biospheres respond differently to environmental and spatial factors in Tibetan hot springs. Frontiers in Microbiology.

