# Supplementary material for: 16S amplicon sequencing of microbial communities in enriched and non-enriched sediments of non-volcanic hot spring with temperature gradients
Source: PeerJ. 2021 Apr 2;9:e10995. doi: 10.7717/peerj.10995 (PMC8020870; doi:10.7717/peerj.10995)
Supplement: Supplemental Information 1 [file peerj-09-10995-s001.docx]

**Supplementary figures and tables**


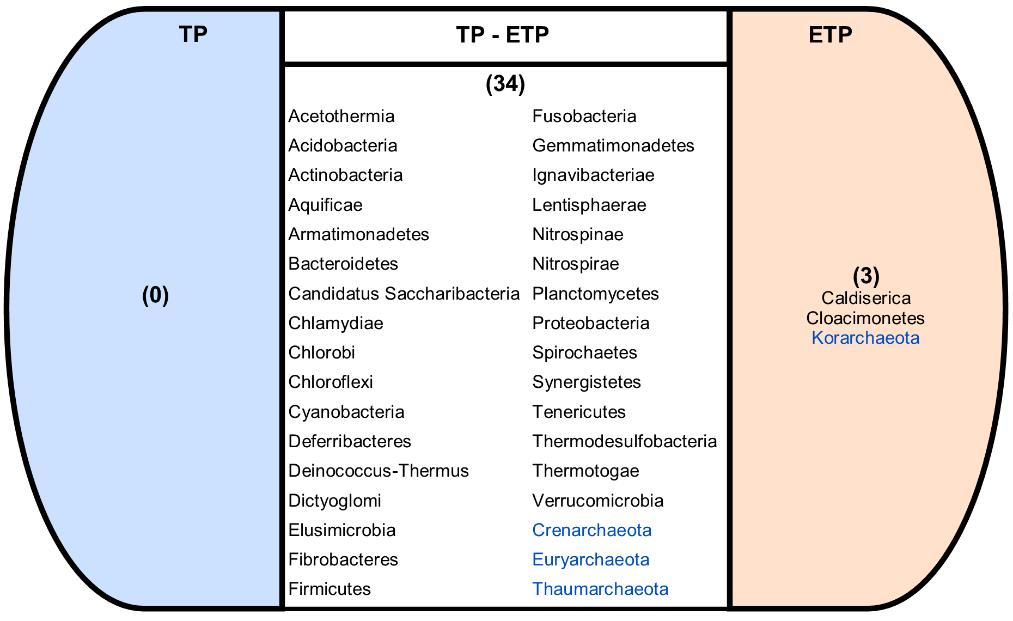


**Fig. S1.** Van diagram of the common and unique bacteria and archaeal phyla between TP and ETP groups. The text in blue color represent archaeal phyla. TP, sediment samples from Tatta Pani hot spring; ETP, enriched sediment samples from Tatta Pani hot spring.


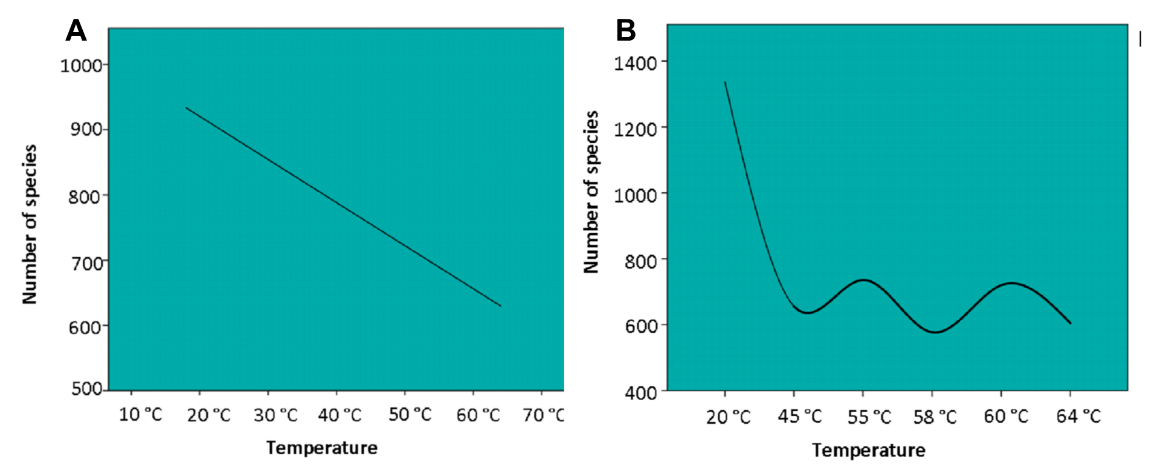


**Fig. S2.** Pearson’s correlation analysis of temperature effect on OTUs diversity at species level **(A)** TP (p=0.003; r -0.8), and **(B)** ETP (p=0.011; r= -0.9). TP, sediment samples from Tatta Pani hot spring; ETP, enriched sediment samples from Tatta Pani hot spring.


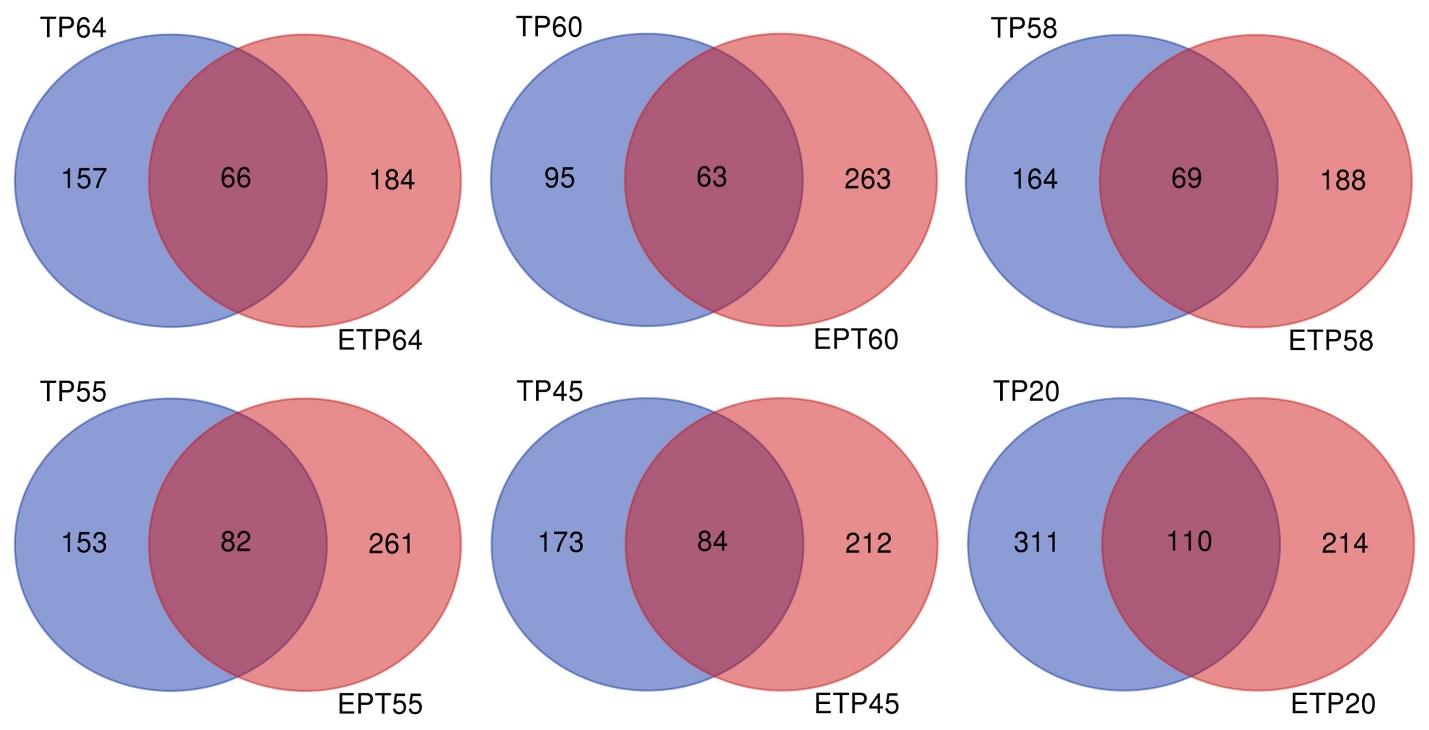


**Fig. S3.** Venn diagram illustrating the distribution of common and unique rare genera OTUs found in less than 0.01% relative abundance between enriched and non-enriched samples collected at different temperatures from the Tatta Pani hot spring. The alphabets in sample name stands for the sample type, and the numeric value represents the temperature in centigrade of the respective sample. TP, sediment samples from Tatta Pani hot spring; ETP, enriched sediment samples from Tatta Pani hot spring.


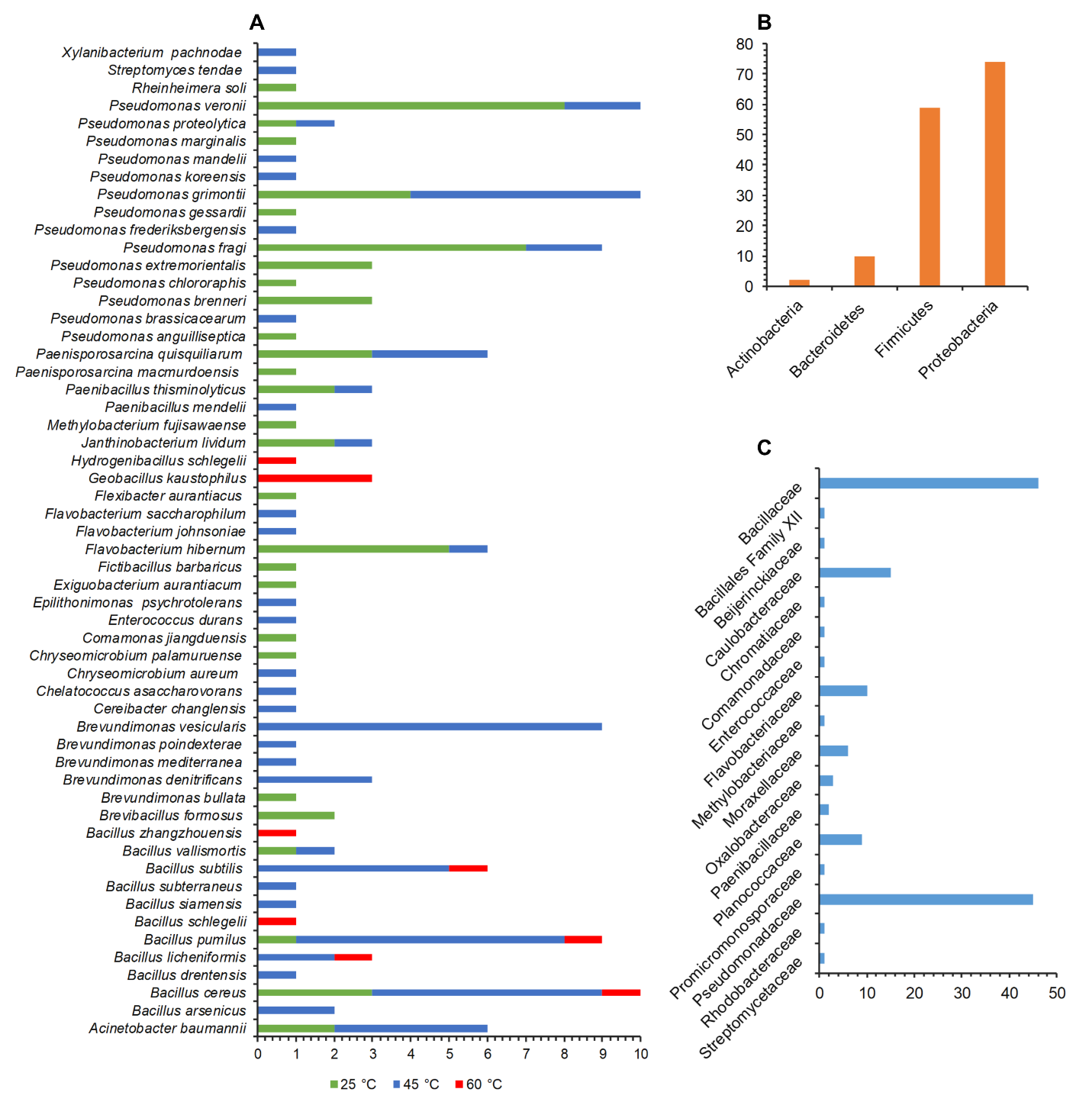


**Fig. S4.** Cultured-dependent analysis of bacterial community from the Tatta Pani hot spring. Abundance of identified (A) species, (B) phyla, and (C) families in number of isolates are showing on x-axis.


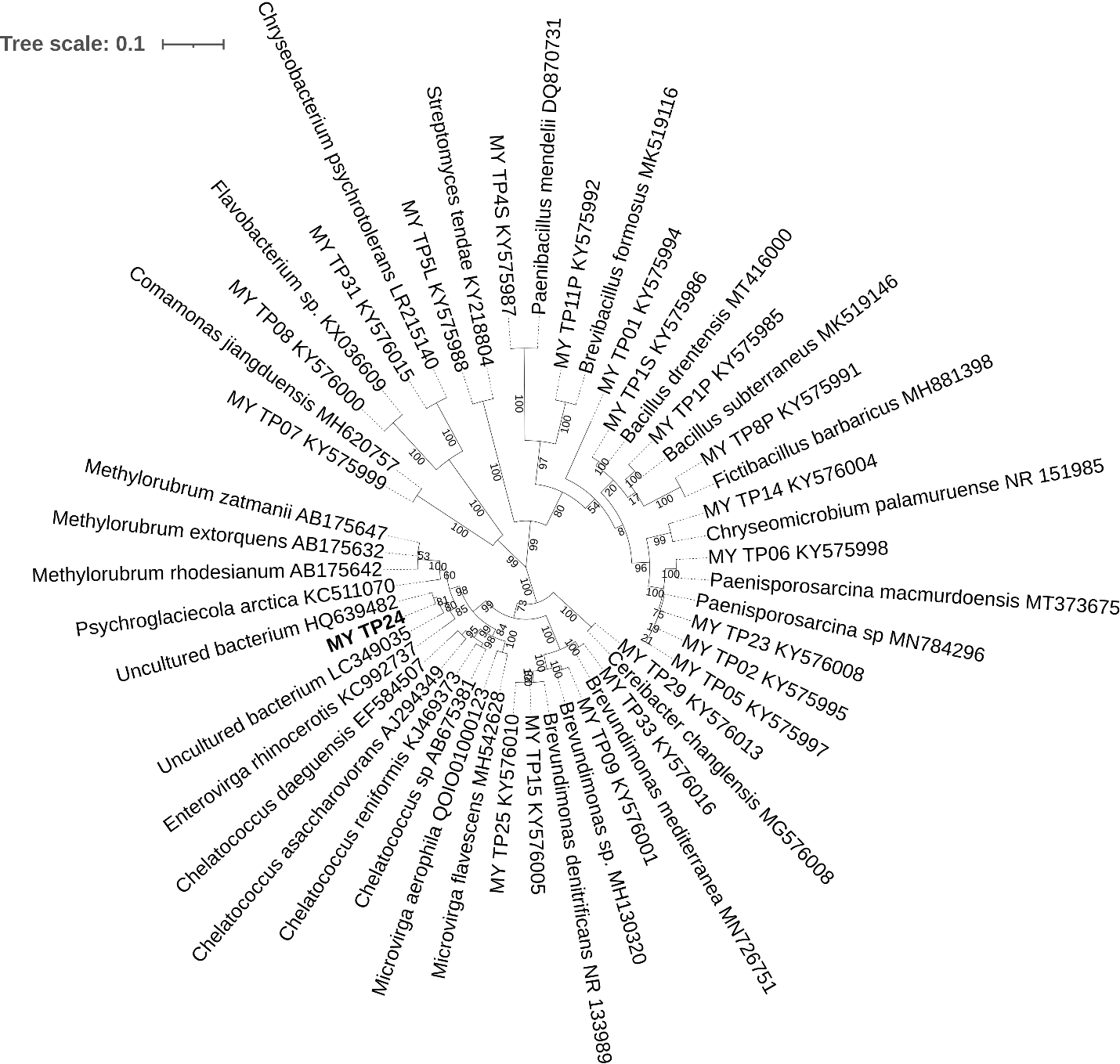


**Fig. S5.** Phylogenetic analysis of 16S rRNA gene sequences showing the relationship of candidate novel isolate MY-TP24 with closely related type strains, and other isolates from the Tatta Pani hot spring. Phylogenetic tree was constructed using Maximum likelihood method with 1000 bootstrap value.

**Table S1.** Samples information collected from the Tatta Pani hot spring.

| Samples ID | Samples Info | Enrichment | Temperature (°C) | pH |
| --- | --- | --- | --- | --- |
| TP64 | Hot spring source | No | 64 | 7.99 |
| TP60 | Down stream | No | 60 | 8.32 |
| TP58 | Down stream | No | 58 | 8.11 |
| TP55 | Down stream | No | 55 | 8.22 |
| TP45 | Down stream | No | 45 | 8.37 |
| TP20 | Down stream | No | 20 | 8.19 |
| ETP64 | Hot spring source | Yes | 64 | 7.99 |
| ETP60 | Down stream | Yes | 60 | 8.32 |
| ETP58 | Down stream | Yes | 58 | 8.11 |
| ETP55 | Down stream | Yes | 55 | 8.22 |
| ETP45 | Down stream | Yes | 45 | 8.37 |
| ETP20 | Down stream | Yes | 20 | 8.19 |

**Table S2.** Media composition of the enrichment culture.

| Composition | Gram/250 ml |
| --- | --- |
| Sodium Chloride | 0.125 |
| Dipotassium Phosphate | 0.06 |
| Casein acid hydrolysate | 0.04 |
| Yeast extract | 0.04 |
| Proteose peptone | 0.04 |
| Dextrose | 0.04 |
| Dipotassium phosphate | 0.025 |
| Magnesium sulphate | 0.003 |
| Sodium pyruvate | 0.025 |
| Distilled water | Make up 125 ml |
| Autoclave | |
| Supplemented at 60 °C | |
| Ascorbic acid | 0.25 |
| Hot spring water 0.2 μm filter sterilized | 125 ml |
| Final volume | 250 ml |

**Table S3.** The number of common phyla identified in samples of TP and ETP groups.

| **Kingdom** | **Group OTUs** | **Common OTUs** | **Samples** | **TP64** | **TP60** | **TP58** | **TP55** | **TP45** | **TP20** | **ETP64** | **ETP60** | **ETP58** | **ETP55** | **ETP45** | **ETP20** |
| --- | --- | --- | --- | --- | --- | --- | --- | --- | --- | --- | --- | --- | --- | --- | --- |
| **Bacteria** | **TP**  **31** | **31** | TP64 | 26 |  |  |  |  |  |  |  |  |  |  |  |
|  |  |  | TP60 | 22 | 25 |  |  |  |  |  |  |  |  |  |  |
|  |  |  | TP58 | 25 | 23 | 27 |  |  |  |  |  |  |  |  |  |
|  |  |  | TP55 | 24 | 22 | 25 | 25 |  |  |  |  |  |  |  |  |
|  |  |  | TP45 | 24 | 22 | 24 | 23 | 24 |  |  |  |  |  |  |  |
|  |  |  | TP20 | 26 | 23 | 27 | 25 | 24 | 29 |  |  |  |  |  |  |
|  |  |  | ETP64 | 26 | 25 | 26 | 25 | 24 | 28 | 30 |  |  |  |  |  |
|  | **ETP**  **33** |  | ETP60 | 25 | 24 | 25 | 24 | 24 | 27 | 28 | 28 |  |  |  |  |
|  |  |  | ETP58 | 24 | 24 | 25 | 24 | 24 | 25 | 26 | 26 | 27 |  |  |  |
|  |  |  | ETP55 | 25 | 23 | 25 | 24 | 23 | 26 | 27 | 26 | 25 | 27 |  |  |
|  |  |  | ETP45 | 24 | 22 | 25 | 24 | 23 | 26 | 26 | 25 | 24 | 25 | 26 |  |
|  |  |  | ETP20 | 26 | 25 | 27 | 25 | 24 | 29 | 30 | 28 | 27 | 27 | 26 | 33 |
|  | **Total = 33** | | | | | | | | | | | | | | |
| **Archaea** | **TP**  **3** | **3** | TP64 | 3 |  |  |  |  |  |  |  |  |  |  |  |
|  |  |  | TP60 | 3 | 3 |  |  |  |  |  |  |  |  |  |  |
|  |  |  | TP58 | 3 | 3 | 3 |  |  |  |  |  |  |  |  |  |
|  |  |  | TP55 | 3 | 3 | 3 | 3 |  |  |  |  |  |  |  |  |
|  |  |  | TP45 | 3 | 3 | 3 | 3 | 3 |  |  |  |  |  |  |  |
|  |  |  | TP20 | 3 | 3 | 3 | 3 | 3 | 4 |  |  |  |  |  |  |
|  | **ETP**  **4** |  | ETP64 | 3 | 3 | 3 | 3 | 3 | 4 | 4 |  |  |  |  |  |
|  |  |  | ETP60 | 3 | 3 | 3 | 3 | 3 | 4 | 4 | 4 |  |  |  |  |
|  |  |  | ETP58 | 3 | 3 | 3 | 3 | 3 | 4 | 4 | 4 | 4 |  |  |  |
|  |  |  | ETP55 | 3 | 3 | 3 | 3 | 3 | 4 | 4 | 4 | 4 | 4 |  |  |
|  |  |  | ETP45 | 3 | 3 | 3 | 3 | 3 | 4 | 4 | 4 | 4 | 4 | 4 |  |
|  |  |  | ETP20 | 3 | 3 | 3 | 3 | 3 | 4 | 4 | 4 | 4 | 4 | 4 | 4 |
|  | **Total = 4** | | | | | | | | | | | | | | |

TP, sediment samples from Tatta Pani hot spring; ETP, enriched sediment samples from Tatta Pani hot spring

**Table S4.** The number of common families identified in samples of TP and ETP groups.

| **Kingdom** | **Group OTUs** | **Common OTUs** | **Samples** | **TP64** | **TP60** | **TP58** | **TP55** | **TP45** | **TP20** | **ETP64** | **ETP60** | **ETP58** | **ETP55** | **ETP45** | **ETP20** |
| --- | --- | --- | --- | --- | --- | --- | --- | --- | --- | --- | --- | --- | --- | --- | --- |
| **Bacteria** | **TP**  **259** | **250** | TP64 | 203 |  |  |  |  |  |  |  |  |  |  |  |
|  |  |  | TP60 | 159 | 173 |  |  |  |  |  |  |  |  |  |  |
|  |  |  | TP58 | 180 | 162 | 206 |  |  |  |  |  |  |  |  |  |
|  |  |  | TP55 | 182 | 159 | 189 | 207 |  |  |  |  |  |  |  |  |
|  |  |  | TP45 | 185 | 161 | 182 | 185 | 204 |  |  |  |  |  |  |  |
|  |  |  | TP20 | 187 | 163 | 195 | 191 | 190 | 228 |  |  |  |  |  |  |
|  | **ETP**  **283** |  | ETP64 | 168 | 151 | 164 | 171 | 167 | 180 | 201 |  |  |  |  |  |
|  |  |  | ETP60 | 176 | 157 | 175 | 180 | 175 | 190 | 180 | 213 |  |  |  |  |
|  |  |  | ETP58 | 167 | 150 | 166 | 169 | 169 | 180 | 175 | 178 | 194 |  |  |  |
|  |  |  | ETP55 | 177 | 153 | 175 | 181 | 175 | 184 | 179 | 190 | 179 | 216 |  |  |
|  |  |  | ETP45 | 168 | 147 | 167 | 169 | 167 | 179 | 173 | 184 | 175 | 184 | 199 |  |
|  |  |  | ETP20 | 195 | 166 | 197 | 198 | 193 | 218 | 193 | 207 | 190 | 204 | 192 | 261 |
|  | **Total = 292** | | | | | | | | | | | | | | |
| **Archaea** | **TP**  **22** | **21** | TP64 | 15 |  |  |  |  |  |  |  |  |  |  |  |
|  |  |  | TP60 | 11 | 15 |  |  |  |  |  |  |  |  |  |  |
|  |  |  | TP58 | 11 | 7 | 11 |  |  |  |  |  |  |  |  |  |
|  |  |  | TP55 | 14 | 12 | 10 | 18 |  |  |  |  |  |  |  |  |
|  |  |  | TP45 | 12 | 12 | 9 | 12 | 15 |  |  |  |  |  |  |  |
|  |  |  | TP20 | 11 | 8 | 10 | 12 | 10 | 12 |  |  |  |  |  |  |
|  | **ETP**  **26** |  | ETP64 | 13 | 10 | 10 | 14 | 11 | 10 | 17 |  |  |  |  |  |
|  |  |  | ETP60 | 16 | 13 | 11 | 17 | 13 | 12 | 17 | 22 |  |  |  |  |
|  |  |  | ETP58 | 16 | 13 | 11 | 18 | 14 | 12 | 17 | 21 | 23 |  |  |  |
|  |  |  | ETP55 | 15 | 12 | 10 | 16 | 12 | 11 | 15 | 20 | 19 | 22 |  |  |
|  |  |  | ETP45 | 15 | 12 | 10 | 16 | 12 | 12 | 16 | 19 | 19 | 18 | 20 |  |
|  |  |  | ETP20 | 16 | 13 | 11 | 16 | 13 | 12 | 17 | 21 | 21 | 20 | 20 | 23 |
|  | **Total = 27** | | | | | | | | | | | | | | |

TP, sediment samples from Tatta Pani hot spring; ETP, enriched sediment samples from Tatta Pani hot spring

**Table S5.** The number of common genera identified in samples of TP and ETP groups.

| **Kingdom** | **Group OTUs** | **Common OTUs** | **Samples** | **TP64** | **TP60** | **TP58** | **TP55** | **TP45** | **TP20** | **ETP64** | **ETP60** | **ETP58** | **ETP55** | **ETP45** | **ETP20** |
| --- | --- | --- | --- | --- | --- | --- | --- | --- | --- | --- | --- | --- | --- | --- | --- |
| **Bacteria** | **TP**  **725** | **609** | TP64 | 454 |  |  |  |  |  |  |  |  |  |  |  |
|  |  |  | TP60 | 302 | 348 |  |  |  |  |  |  |  |  |  |  |
|  |  |  | TP58 | 354 | 296 | 462 |  |  |  |  |  |  |  |  |  |
|  |  |  | TP55 | 369 | 294 | 394 | 484 |  |  |  |  |  |  |  |  |
|  |  |  | TP45 | 375 | 299 | 383 | 395 | 489 |  |  |  |  |  |  |  |
|  |  |  | TP20 | 393 | 311 | 407 | 426 | 430 | 589 |  |  |  |  |  |  |
|  |  |  | ETP64 | 273 | 232 | 273 | 282 | 284 | 318 | 428 |  |  |  |  |  |
|  | **ETP**  **890** |  | ETP60 | 295 | 250 | 307 | 313 | 312 | 352 | 337 | 500 |  |  |  |  |
|  |  |  | ETP58 | 269 | 220 | 261 | 275 | 278 | 303 | 312 | 339 | 405 |  |  |  |
|  |  |  | ETP55 | 303 | 251 | 308 | 317 | 319 | 347 | 338 | 385 | 336 | 505 |  |  |
|  |  |  | ETP45 | 278 | 266 | 281 | 294 | 288 | 322 | 315 | 359 | 312 | 360 | 444 |  |
|  |  |  | ETP20 | 389 | 307 | 398 | 411 | 414 | 505 | 394 | 461 | 378 | 453 | 409 | 778 |
|  | **Total = 1006** | | | | | | | | | | | | | | |
| **Archaea** | **TP**  **31** | **29** | TP64 | 21 |  |  |  |  |  |  |  |  |  |  |  |
|  |  |  | TP60 | 15 | 21 |  |  |  |  |  |  |  |  |  |  |
|  |  |  | TP58 | 14 | 10 | 15 |  |  |  |  |  |  |  |  |  |
|  |  |  | TP55 | 18 | 15 | 13 | 25 |  |  |  |  |  |  |  |  |
|  |  |  | TP45 | 17 | 16 | 13 | 17 | 20 |  |  |  |  |  |  |  |
|  |  |  | TP20 | 14 | 10 | 12 | 15 | 13 | 16 |  |  |  |  |  |  |
|  | **ETP**  **51** |  | ETP64 | 16 | 13 | 12 | 18 | 14 | 11 | 23 |  |  |  |  |  |
|  |  |  | ETP60 | 18 | 16 | 12 | 21 | 16 | 12 | 22 | 32 |  |  |  |  |
|  |  |  | ETP58 | 18 | 16 | 12 | 22 | 17 | 12 | 21 | 27 | 34 |  |  |  |
|  |  |  | ETP55 | 18 | 16 | 13 | 21 | 16 | 13 | 21 | 27 | 27 | 34 |  |  |
|  |  |  | ETP45 | 16 | 14 | 10 | 19 | 14 | 12 | 20 | 25 | 27 | 24 | 34 |  |
|  |  |  | ETP20 | 18 | 16 | 13 | 20 | 15 | 13 | 20 | 26 | 27 | 27 | 26 | 33 |
|  | **Total = 53** | | | | | | | | | | | | | | |

TP, sediment samples from Tatta Pani hot spring; ETP, enriched sediment samples from Tatta Pani hot spring

**Table S6.** The number of common OTUs at species level identified in samples of TP and ETP groups.

| **Kingdom** | **Group OTUs** | **Common OTUs** | **Samples** | **TP64** | **TP60** | **TP58** | **TP55** | **TP45** | **TP20** | **ETP64** | **ETP60** | **ETP58** | **ETP55** | **ETP45** | **ETP20** |
| --- | --- | --- | --- | --- | --- | --- | --- | --- | --- | --- | --- | --- | --- | --- | --- |
| **Bacteria** | **TP**  **1192** | **843** | TP64 | 662 |  |  |  |  |  |  |  |  |  |  |  |
|  |  |  | TP60 | 400 | 486 |  |  |  |  |  |  |  |  |  |  |
|  |  |  | TP58 | 450 | 375 | 650 |  |  |  |  |  |  |  |  |  |
|  |  |  | TP55 | 487 | 385 | 527 | 709 |  |  |  |  |  |  |  |  |
|  |  |  | TP45 | 501 | 401 | 497 | 525 | 709 |  |  |  |  |  |  |  |
|  |  |  | TP20 | 527 | 410 | 542 | 592 | 589 | 908 |  |  |  |  |  |  |
|  |  |  | ETP64 | 303 | 254 | 284 | 301 | 313 | 337 | 605 |  |  |  |  |  |
|  | **ETP**  **1648** |  | ETP60 | 320 | 277 | 323 | 340 | 344 | 382 | 422 | 720 |  |  |  |  |
|  |  |  | ETP58 | 293 | 244 | 237 | 298 | 303 | 316 | 395 | 438 | 577 |  |  |  |
|  |  |  | ETP55 | 316 | 264 | 316 | 337 | 338 | 370 | 413 | 506 | 422 | 736 |  |  |
|  |  |  | ETP45 | 297 | 247 | 282 | 317 | 310 | 346 | 392 | 466 | 399 | 467 | 656 |  |
|  |  |  | ETP20 | 461 | 360 | 472 | 511 | 500 | 645 | 516 | 630 | 493 | 617 | 542 | 1336 |
|  | **Total = 1997** | | | | | | | | | | | | | | |
| **Archaea** | **TP**  **44** | **38** | TP64 | 29 |  |  |  |  |  |  |  |  |  |  |  |
|  |  |  | TP60 | 19 | 23 |  |  |  |  |  |  |  |  |  |  |
|  |  |  | TP58 | 18 | 12 | 20 |  |  |  |  |  |  |  |  |  |
|  |  |  | TP55 | 25 | 18 | 18 | 35 |  |  |  |  |  |  |  |  |
|  |  |  | TP45 | 22 | 18 | 17 | 22 | 26 |  |  |  |  |  |  |  |
|  |  |  | TP20 | 18 | 12 | 15 | 20 | 16 | 24 |  |  |  |  |  |  |
|  | **ETP**  **80** |  | ETP64 | 16 | 13 | 12 | 15 | 13 | 9 | 28 |  |  |  |  |  |
|  |  |  | ETP60 | 23 | 19 | 16 | 28 | 19 | 17 | 21 | 46 |  |  |  |  |
|  |  |  | ETP58 | 22 | 20 | 15 | 25 | 18 | 16 | 21 | 32 | 44 |  |  |  |
|  |  |  | ETP55 | 22 | 18 | 15 | 26 | 18 | 16 | 24 | 37 | 34 | 50 |  |  |
|  |  |  | ETP45 | 18 | 16 | 13 | 21 | 15 | 16 | 20 | 31 | 31 | 30 | 43 |  |
|  |  |  | ETP20 | 24 | 20 | 16 | 26 | 18 | 18 | 22 | 33 | 34 | 36 | 32 | 50 |
|  | **Total = 86** | | | | | | | | | | | | | | |

TP, sediment samples from Tatta Pani hot spring; ETP, enriched sediment samples from Tatta Pani hot spring
